# Synthesis of Branched α‐Olefins via Trimerization and Tetramerization of Ethylene

**DOI:** 10.1002/advs.202405653

**Published:** 2024-08-09

**Authors:** Fabian Lukas, Paula A. Simon, Thomas Dietel, Winfried P. Kretschmer, Rhett Kempe

**Affiliations:** ^1^ Lehrstuhl für Anorganische Chemie II – Katalysatordesign Sustainable Chemistry Centre University of Bayreuth 95440 Bayreuth Germany

**Keywords:** branched α‐olefins, ethylene, tetramerization, titanium, trimerization

## Abstract

α‐Olefins are very important bulk and fine chemicals and their synthesis from ethylene, an abundantly available and inexpensive feedstock, is highly attractive. Unfortunately, the direct or on‐purpose synthesis of olefins from ethylene is limited to three examples, 1‐butene, 1‐hexene, and 1‐octene, all having a linear structure. Herein, the direct synthesis of 3‐methylenepentane and 4‐ethylhex‐1‐ene, branched trimerization, and tetramerization products of ethylene, respectively, is reported. Different molecular titanium catalysts, all highly active, with a selectivity toward the formation of the branched ethylene trimer or tetramer, the employment of different activators, and different reaction conditions are the key to selective product formation. The long‐time stability of selected catalysts employed permits upscaling as demonstrated for the synthesis of 4‐ethylhex‐1‐ene (52 g isolated, TON(ethylene) 10.7 · 10^6^).

## Graphical abstract

The direct syntheses of 3‐methylenepentane and 4‐ethylhex‐1‐ene from ethylene are introduced. The key to the novel ethylene trimerization and tetramerization reactions are molecular titanium catalysts with a high selectivity toward the formation of one or other branched α‐olefin, different activators, and different reaction conditions employed.

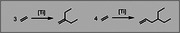

## 1 Introduction

**Figure 1:**
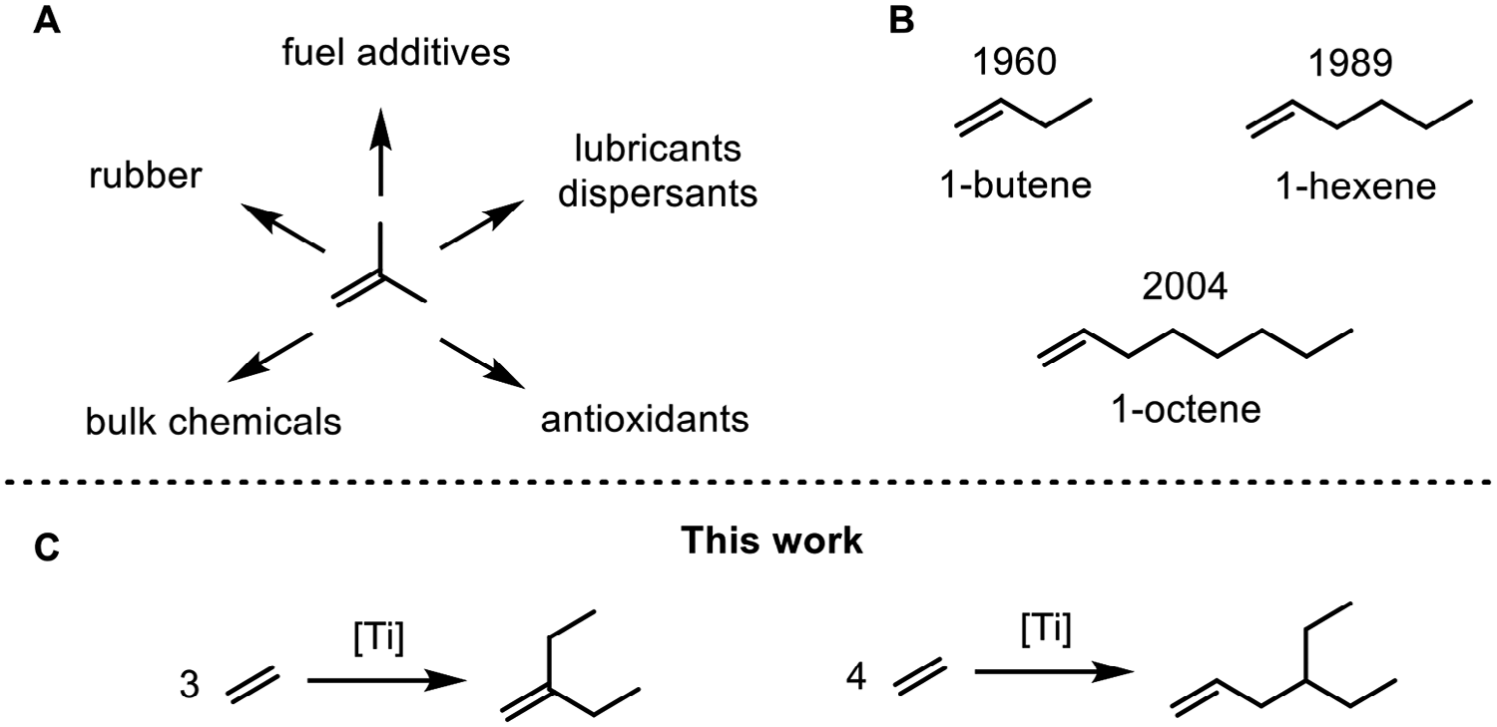
State of the art and work reported here. A) Commercial products of isobutylene. B) On‐purpose α‐olefin syntheses from ethylene and date of their initial disclosure. C) Ethylene trimerization toward 3MP and tetramerization toward 4EH reported here.

α‐Olefins are produced on megaton scale and used for the production of plastics and lubricants or as a starting material in important functionalization reactions.^[^ ^1^ ^]^ Ethylene is an attractive feedstock for the synthesis or production of α‐olefins, since it is abundantly available, also from renewable resources,^[^ ^2^ ^]^ and inexpensive. The production of α‐olefins from ethylene is one of the largest applications of homogeneous catalysis and leads to product distributions (for instance a *Schulz‐Flory* ^[^ ^3^ ^]^ distribution) or for a few examples to direct or on‐purpose products.^[^ ^1^ ^]^ The direct or on‐purpose synthesis is greener or more sustainable, since it reduces by‐product formation drastically. An example of an important branched α‐olefin is 2‐methylprop‐1‐ene or isobutylene (**Figure** **1**A). It is used to produce rubbers, bulk chemicals, lubricants, dispersants, fuel additives and antioxidants (Figure 1A).^[^ ^1^ ^]^ The linear α‐olefins, which are directly accessible from ethylene (Figure 1B): 1‐butene,^[^ ^4^ ^]^ 1‐hexene^[^ ^5^ ^]^ and 1‐octene,^[^ ^6^ ^]^ introduced in 1960, 1989, and 2004, respectively,^[^ ^7^ ^]^ are mostly used as co‐feed for polyolefin plastic production and as starting materials for bulk chemical syntheses. We introduced the elongation and branching of α‐olefins by two ethylene molecules^[^ ^8^ ^]^ and investigated the potential of Zr catalysts for such reactions recently.^[^ ^9^ ^]^ The Zr catalysts showed less selectivity in comparison to Ti catalysts and we decided to further work with Ti catalysts. Here we report the direct synthesis of 3‐methylenepentane (3MP) and 4‐ethylhex‐1‐ene (4EH) from ethylene (Figure 1C). 3MP is a branched ethylene trimerization product structurally and functionally related to isobutylene and 4EH is a branched α‐olefin related with regard to accessibility and function to 1‐butene, 1‐hexene and 1‐octene or the propylene dimerization product 4‐methylpent‐1‐ene.^[^ ^10^ ^]^ 3MP is observed as a by‐product in the Alphabutol process.^[^ ^11^ ^]^ In addition, other ethylene dimerization catalysts generate this by‐product too.^[^ ^12^ ^]^ 4EH has not been observed in these processes yet. A multi‐step synthesis based on catalytic organic synthesis involving steps such as hydroformylation, hydrogenation, ester formation and decarboxylation was reported.^[^ ^13^ ^]^

Different molecular Ti catalysts with a high selectivity toward the formation of the branched ethylene trimer or tetramer, the employment of different activators and different reaction conditions are the key to selective syntheses of 3MP or 4EH. The long‐time stability of selected catalysts employed permits upscaling as demonstrated for the synthesis of 4EH (52 g isolated, TON(ethylene): 10.7 · 10^6^) in a 1 L autoclave (see Table S15, Supporting Information).

## 2 Results and Discussion

**Figure 2:**
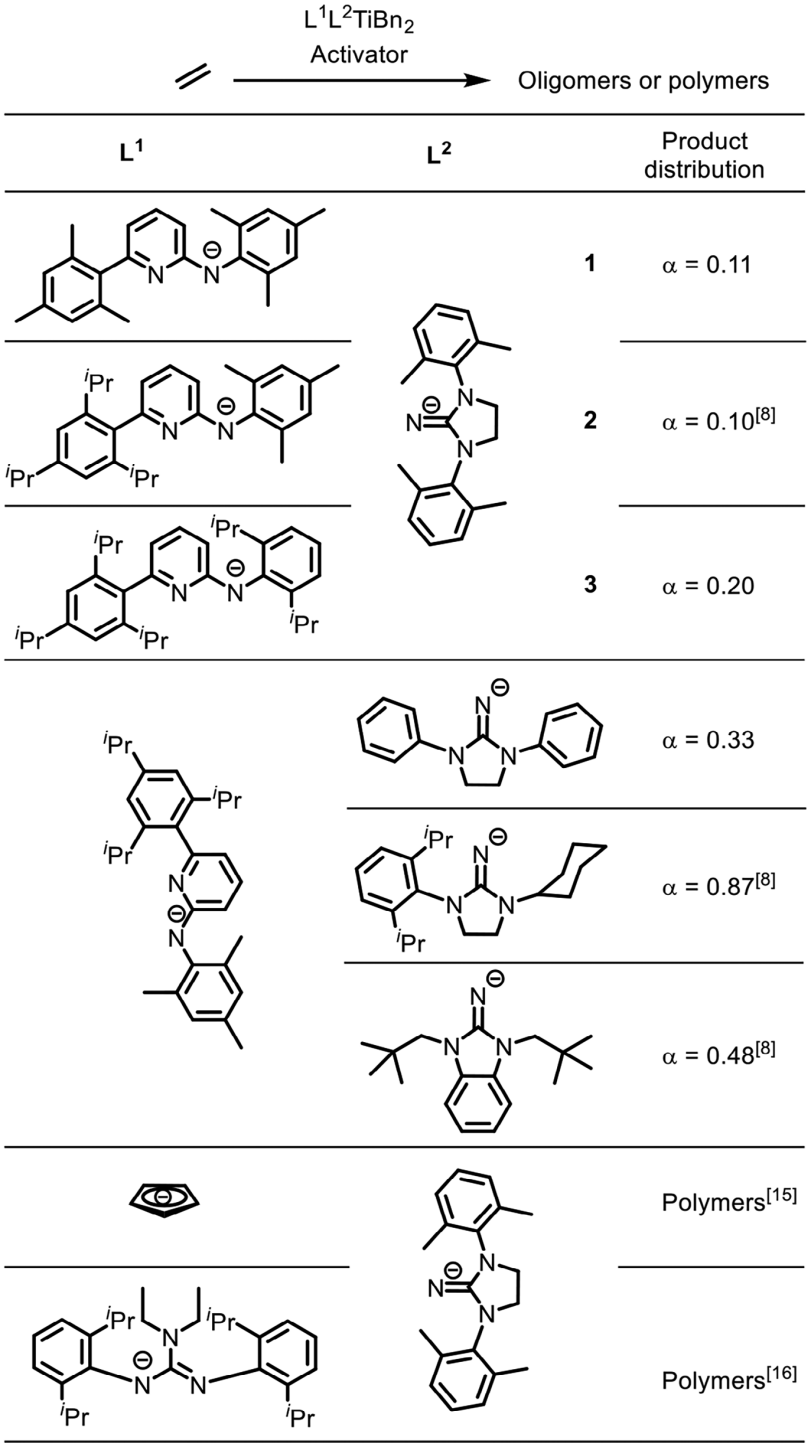
Variation of the ancillary ligands L^1^ and L^2^ of L^1^L^2^Ti ethylene oligomerization or polymerization catalysts and the resulting product distribution obtained (Bn = benzyl).

The investigation of the α‐value^[^ ^14^ ^]^ of numerous Ti complexes revealed that only bulky aminopyridinato (Ap) and specific (imidazolidine‐2‐ylidene)amido (Imi) ligand combinations permit very fast chain termination in the homo‐oligomerization of ethylene (**Figure** **2**). Note that the α‐value of the ethylene homo‐oligomerization and the α‐value of the α‐olefin‐ethylene co‐oligomerization are identical and very low α‐values are crucial for observing selective co‐oligomerization reactions.^[^ ^8^ ^]^ The activated L^1^L^2^TiBn_2_ (L^1^, L^2^: ancillary ligands; Bn: benzyl) precatalysts were used as catalysts in the oligomerization/polymerization of ethylene (room temperature and 1.7 bar ethylene pressure; reaction conditions see Table S3, Supporting Information).

Starting with deprotonated N,6‐dimesitylpyridin‐2‐amine (Ap^6Me^), which contains two mesitylene substituents, and deprotonated 1,3‐bis(2,6‐dimethylphenyl)imidazolidin‐2‐imine (Imi^Me^) as the ancillary ligands (precatalyst 1), an α‐value of 0.11 (corresponds to 89 mol‐% 1‐butene) was observed. The change of the pyridyl substituent to a bulkier 2,4,6‐triisopropylphenyl group (deprotonated N‐mesityl‐6‐(2,4,6‐triisopropylphenyl)pyridin‐2‐amine = Ap^9Me^, precatalyst 2)^[^ ^8^ ^]^ yielded a similar product distribution with α  =  0.10, whereas further substitution of the amido aryl group to 2,6‐diisopropylphenyl (deprotonated N‐(2,6‐diisopropylphenyl)−6‐(2,4,6‐triisopropylphenyl)pyridin‐2‐amine = Ap*, precatalyst 3) resulted in a lower 1‐butene selectivity with α  =  0.20. Further variations of the Imi ligand with Ap^9Me^ as the second ancillary ligand led to oligomers with generally too high α‐values (between 0.33 and 0.87). A shift to polymeric products was observed when replacing the Ap ligand with a cyclopentadienyl or a η^2^‐N‐donor guanidinato ligand.^[^ ^15^, ^16^ ^]^ With a cut‐off value of α  =  0.2 for further studies, precatalysts 1, 2 and 3 seemed to be potentially suitable candidates for the ethylene trimerization and tetramerization toward branched α‐olefin products.

**Figure 3:**
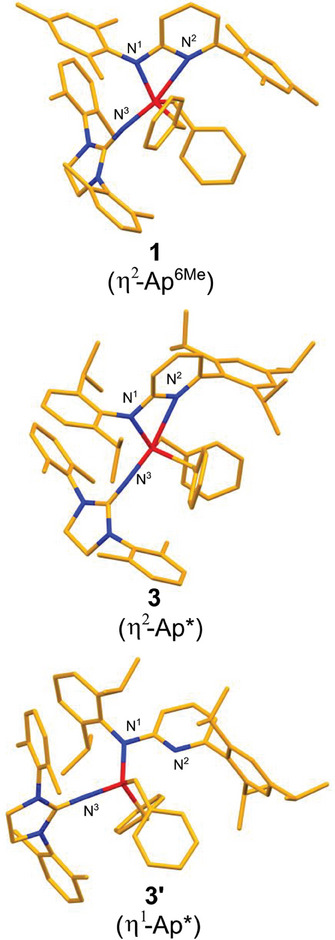
Molecular structures of 1, 3, and 3′. X‐ray single crystal analysis for complex 3 reveals two independent and chemically different molecules in the asymmetric unit – η^2^ coordination of the aminopyridinato ligand in 3 and η^1^ in 3′. Selected bond lengths: Ti‐N^1^: 1: 2.010(6) Å; 3: 2.021(3) Å; 3′: 1.979(3) Å; Ti‐N^2^: 1: 2.456(7) Å; 3: 2.534(3) Å; 3′:/; Ti‐N^3^: 1: 1.812(8) Å; 3: 1.806(3) Å; 3′: 1.816(3) Å. For plots that indicate the steric overload, see Figure S4 (Supporting Information).

X‐ray single crystal analysis for complexes **1** and **3** (η^2^ coordinated Ap*) reveal a distorted trigonal bipyramidal coordination (**Figure** **3**). In both cases, the axial positions are occupied by the pyridyl‐N^2^ and Imi‐N^3^ atoms. It is noteworthy that complex **3** with the sterically very demanding Ap* ligand exhibits an asymmetric unit with a second independent molecule **3′** (η^1^ coordinated Ap*), which has a distorted tetrahedral coordination. The Ti‐amido‐N^1^ bond lengths do not differ significantly between the three structures with about 2.0 Å and are comparable to typical mono‐Ap titanium complexes. The Ti‐pyridyl‐N^2^ bond lengths, on the other hand, are rather long with about 2.5 Å compared to an averaged 2.2 Å found in other Ap‐Ti complexes.^[^ ^17^ ^]^ This may be attributed to a generally densely packed titanium coordination sphere. The Ti‐Imi‐N^3^ bond lengths are about 1.8 Å and similar to previously reported Imi‐Ti complexes.^[^ ^15^ ^]^

**Figure 4:**
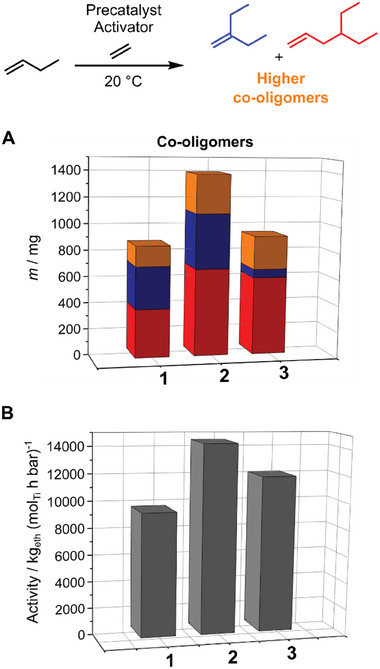
Co‐oligomerization of 1‐butene and ethylene catalyzed by complexes **1**, **2,** or **3** after activation with ammonium borate [R_2_N(CH_3_)H]^+^[B(C_6_F_5_)_4_]^−^ (R  =  C_16_H_33_ to C_18_H_37_). Note that 1‐butene has been added. Comparison of the co‐oligomer mass distribution A) and the activity B). Reaction conditions: *p*(eth)  =  1.7 bar; *T*  =  20 °C; *V*(eth)  =  1 L_n_; *n*(Ti)  =  0.1 µmol; Ti/B  =  1/1.1; *n*(1‐butene)  =  50 mmol; solvent is methylcyclohexane; *V*(sum)  =  15 mL; scavenger  =  30 µmol triisobutylaluminum (TIBA). Color code: red  =  4EH, blue  =  3MP and orange  =  higher molecular weight co‐oligomers.

The ethylene trimerization and tetramerization toward branched olefins formally consist of the ethylene dimerization to 1‐butene as the first step and the subsequent co‐oligomerization with ethylene toward the trimerization product 3MP or the tetramerization product 4EH as the second step. To determine the most selective precatalyst for either product, we investigated the elongation/branching reaction of 1‐butene with ethylene (**Figure** **4**). 1‐Butene (50 mmol) in methylcyclohexane as the solvent (15 mL sum) were reacted with 1 L_n_ of ethylene at 1.7 bar in the presence of the activated precatalyst at 20 °C (for full experimental details see Table S5, Supporting Information). The co‐oligomer product distribution and total amount of co‐oligomers produced differed significantly depending on the precatalyst's structure (Figure 4A). The catalyst system based on **1** with the sterically smallest Ap ligand (Ap^6Me^) produced the highest 3MP content with 46 mol‐%. Increasing the sterical bulk led to lower co‐dimer formation with 38 mol‐% for precatalyst **2** (Ap^9Me^) and 10 mol‐% for complex **3** (Ap*). The inverse trend was observed for the 4EH selectivity and the highest percentage of 69 mol‐% was achieved with **3**. The total co‐oligomer mass or co‐oligomer productivity was also influenced by the precatalyst's structure. Complex **2** exhibited the highest α‐olefin response with 1.4 g (71 wt‐%), whereas **1** and **3** were similar with about 0.9 g (51 wt‐% and 54 wt‐% respectively). The remaining ethylene was oligomerized to *Schulz‐Flory* distributed linear α‐olefins. All three catalytic systems were highly active and showed activities between 10 000 and 14 000 kg(ethylene)/(mol(Ti) h bar) (Figure 4B). Precatalyst **1** was chosen for investigating 3MP formation (ethylene trimerization), while **3** was the most promising precatalyst for the formation of 4EH (ethylene tetramerization).

The direct ethylene trimerization reaction was investigated varying reaction temperature, activator and ethylene pressure applied (**Table** **1**). A starting solvent volume of 10 mL (methylcyclohexane) was chosen to allow for a rapid 1‐butene buildup at relatively low ethylene conversion. Low temperatures are favorable for the trimer formation and the highest 3MP content of 58 mol‐% was achieved at −10 °C with 25 mol‐% 4EH as the major by‐product and 10 mol‐% of both ethylene pentamers combined. Of these pentamers, 4‐ethyl‐1‐octene (4EO) represents the second most important hetero‐oligomer by‐product with a share of 9 mol‐%. Its origin lies in two different reaction pathways. One the one hand, it arises as a pentamer of ethylene from the reaction of 1‐butene with three molecules of ethylene. On the other hand, it is the hetero‐trimer of 1‐hexene and two ethylene molecules. The latter reaction is problematic because 1‐hexene is produced alongside 1‐butene as part of the linear α‐olefin *Schulz‐Flory* distribution. Changing the activator from an anilinium borate type ([R_2_N(CH_3_)H]^+^[B(C_6_F_5_)_4_]^−^; R  =  C_16_H_33_ to C_18_H_37_) to trimethylaluminum‐depleted methylalumoxane (d‐MAO) under identical reaction conditions resulted in a shift to longer products. Generally, we observe that borate activation is more beneficial for trimerization (see Table S10, Supporting Information, Entries 16 and 17). We assume that the various anion^[^ ^18^ ^]^ sizes of the activator lead to a different inter‐ion interaction resulting in different ethylene binding and selectivity. Higher ethylene pressure led to more long‐chain products, whereas the trimer percentage decreased. This trend is in agreement with an increase of the system's α‐value (α‐values see Table S10, Supporting Information). As a result, a higher tendency to insert more ethylene molecules, before chain termination occurs, is expected.

**Table 1:** Influence of reaction temperature, activator and ethylene pressure on the selectivity of the direct ethylene trimerization using precatalyst **1**. The selectivity of the co‐oligomers is shown.^a^ ^)^ We observe a higher α‐value for both processes co‐oligomerization and formation of linear α‐olefins at higher temperatures. These two α‐values are identical.

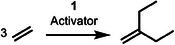							
Entry	Activator	*T*/°C	*p*(eth)/bar	Co‐oligomers/mol‐%			
				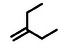	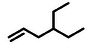	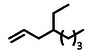	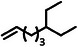
1	borate	−10	0.9^b)^	58	25	9	1
2	borate	0	1.2^b)^	55	28	10	1
3	borate	20	1.7^b)^	40	33	14	2
4	d‐MAO	20	1.7	35	36	16	2
5	borate	20	3.0	29	37	19	3

^a)^ Reaction conditions: *n*(Ti)  =  300 nmol; activator: $\frac{n\left(d-\mathrm{MAO}\right)}{n\left(\mathrm{Ti}\right)}\;=\;250$ (d‐MAO: trimethylaluminum‐depleted methylalumoxane) or $\frac{n\left(\mathrm{borate}\right)}{n\left(\mathrm{Ti}\right)}\;=\;1.1$; scavenger: *n*(TIBA)  =  30 µmol; *V*(methylcyclohexane)  =  10 mL; *V*(eth) = 1 L_n_; internal standard: cumene; selectivity based on hetero‐oligomer distribution;

^b)^ *c*(eth)  =  const.

Our direct ethylene tetramerization was investigated in a similar fashion using a catalytic system based on **3** (**Table** **2**). Compared to the previous study, usage of d‐MAO as the activator proved more beneficial for 4EH formation than the borate activator with 58 mol‐% compared to 55 mol‐% at 20 °C and 1.7 bar ethylene (see also Table S12, Supporting Information, Entries 28 and 29). The highest selectivity of 66 mol‐% toward the desired tetramer was achieved at −10 °C. The major by‐products consisted of the ethylene trimer 3MP (13 mol‐%) and pentamer 4EO (14 mol‐%). An increase in temperature as well as ethylene pressure led to a decrease in the 4EH content in favor of longer‐chain products, which can also be explained by a higher α‐value (α‐values see, Table S12, Supporting Information).

**Table 2:** Influence of reaction temperature, activator and ethylene pressure on the selectivity of the direct ethylene tetramerization using precatalyst **3**. The selectivity of the co‐oligomers is shown.^a^ ^)^

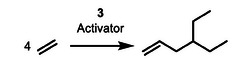							
Entry	Activator	*T*/°C	*p*(eth)/bar	Co‐oligomers/mol‐%			
				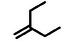	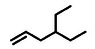	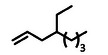	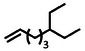
6	d‐MAO	−10	0.9^b^ ^)^	13	66	14	3
7	d‐MAO	0	1.2^b^ ^)^	10	64	16	4
8	borate	20	1.7	9	55	18	7
9	d‐MAO	20	1.7^b^ ^)^	7	58	20	6
10	d‐MAO	20	3.0	5	48	21	8

^a)^ Reaction conditions: *n*(Ti)  =  300 nmol; $\frac{n\left(d-\mathrm{MAO}\right)}{n\left(\mathrm{Ti}\right)}\;=\;250$ or $\frac{n\left(\mathrm{borate}\right)}{n\left(\mathrm{Ti}\right)}\;=\;1.1$; scavenger: *n*(TIBA)  =  30 µmol; *V*(methylcyclohexane)  =  10 mL; *V*(eth) = 1 L_n_; internal standard: cumene; selectivity based on hetero‐oligomer distribution;

^b)^ *c*(eth)  =  const.

Finally, we performed a larger‐scale experiment in a water‐cooled 1 L steel autoclave for the synthesis of 4EH using complex **3** as precatalyst (**Table** **3**). We used 1‐butene as the solvent to minimize by‐product formation. The experiment was carried out at 30 °C to enable sufficient cooling capacity during the runtime and we chose 4.0 bar of ethylene pressure due to 1‐butene vapor pressure. Samples were taken during the reaction to follow the co‐oligomer distribution (Table 3, Entries 11–13). The catalyst system displayed excellent long‐term stability with an activity of 9100 kg(eth)/(mol(Ti) h bar) even after 100 L_n_ of reacted ethylene. A continuously high 1‐butene concentration during the reaction is key to enable upscaling without the drastic loss of selectivity toward the desired product. A 4EH selectivity of 76 mol‐% (10 L_n_ ethylene) was achieved which slightly decreased to 68 mol‐% after 100 L_n_ of ethylene consumption. Based on the catalyst's ethylene homo‐oligomerization behavior, the 1‐butene consumed was compensated and more 1‐butene was formed than originally added—1.1 g after reacting 10 L_n_ of ethylene for instance. The 1‐butene formed and originally added can be distilled off after the reaction and reused. The crude reaction product could be purified via a single step distillation (eight sections Snyder distillation column) and was performed for Entry 37, Table S15 (Supporting Information, 52 g isolated yield, 93 wt%, TON(ethylene) 10.7 10^6^). See Table S16 (Supporting Information) for a larger‐scale formation of 3MP.

**Table 3:** Large‐scale synthesis of 4EH using precatalyst **3**. The selectivity of the co‐oligomers is shown.^a)^

Entry	*V*(eth)/L_n_	Co‐oligomers/mol‐%		
		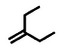	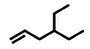	Higher co‐oligomers
11	10	7 (0.4 g)	76 (6.5 g)	17
12	50	7 (1.9 g)	71 (26.0 g)	22
13	100	7 (3.8 g)	68 (47.8 g)	25

^a)^ Reaction conditions: *n*(Ti)  =  500 nmol; activator: d‐MAO $\frac{n(d-\mathrm{MAO})}{n(\mathrm{Ti})}\;=\;250$; scavenger: *n*(TIBA)  =  200 µmol; *V*(1‐butene)  =  200 mL; *T*  =  30 °C; *p*  =  4.0 bara; internal standard: cumene; selectivity based on co‐oligomer distribution; activity = 9100$\frac{\mathrm{kg}(\mathrm{eth})}{\mathrm{mol}(\mathrm{Ti})\cdot h\cdot \mathrm{bar}}$.

**Scheme 1:**
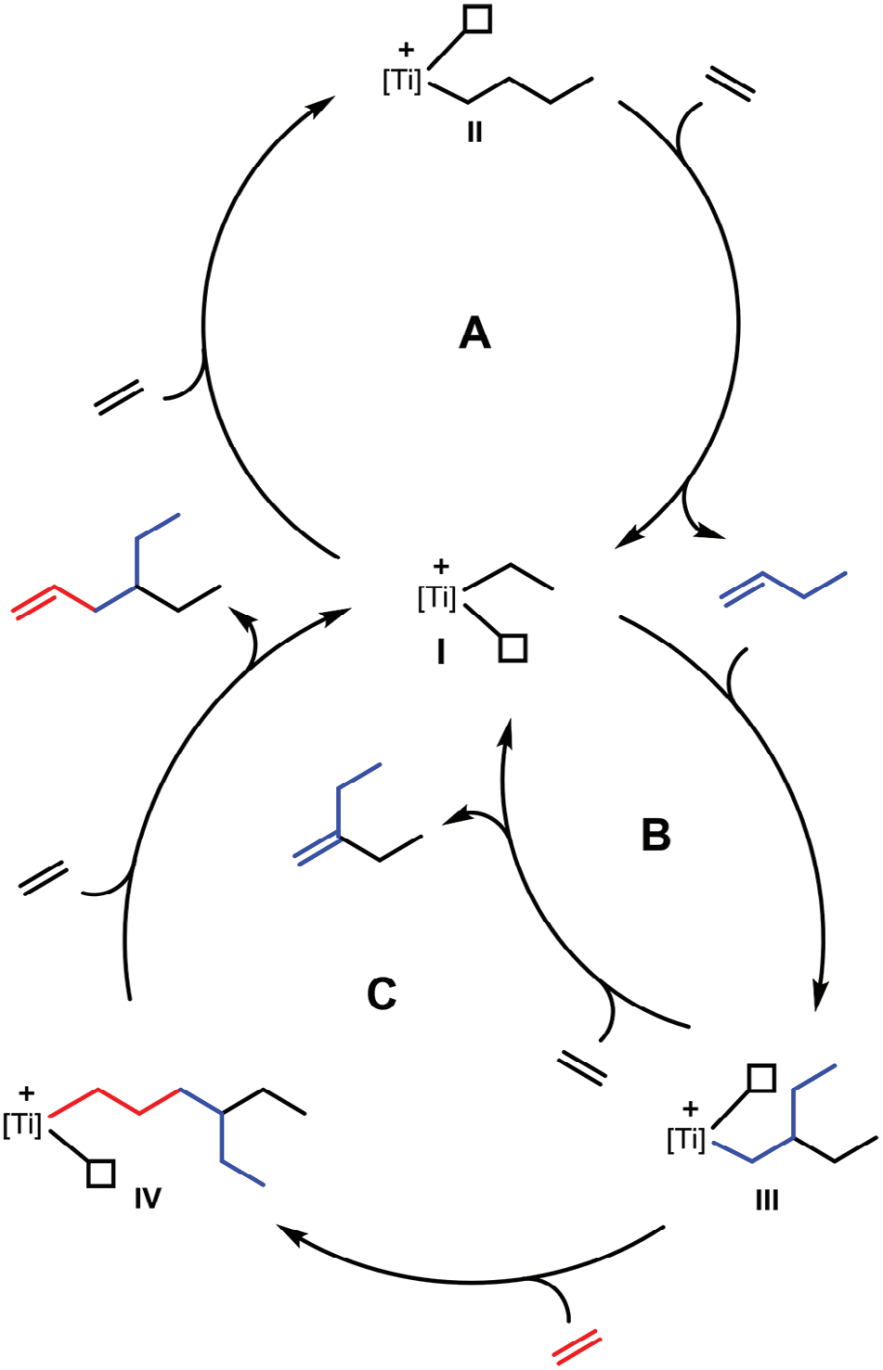
Mechanism proposed with A) 1‐butene cycle, B) 3MP cycle, and C) 4EH cycle.

We propose a *Cossee*‐*Arlman* type insertion mechanism for our titanium catalysts (**Scheme** **1**).^[^ ^8^ ^]^ The titanium ethanido intermediate I is the starting point for the dimerization cycle A and the co‐oligomerization cycles B and C. The insertion of one ethylene molecule yields intermediate II and β‐H‐elimination/transfer to ethylene completes the first cycle by releasing 1‐butene. However, intermediate I can also undergo a regioselective 1,2‐insertion of 1‐butene to form intermediate III. The ß‐H elimination/transfer to ethylene at this stage releases 3MP and completes cycle B, whereas the further reaction with one molecule ethylene leads to intermediate IV. The starting intermediate I is restored by forming 4EH in an ß‐H elimination/transfer reaction with ethylene, thus, completing the third cycle C. Interestingly, products which result from a 2,1‐insertion and other chain‐termination reactions are not observed.

## 3 Conclusion

In conclusion, we introduced the direct synthesis of 3MP, an ethylene trimerization product, and the direct synthesis of 4EH, an ethylene tetramerization product. The key is employing different highly active Ap‐Imi titanium catalysts with a selectivity toward the formation of one or the other α‐olefin, the employment of different activators, ammonium borate for the trimerization and d‐MAO for the tetramerization product, and different optimized reaction conditions. The long‐time stability of selected catalysts employed permits upscaling as demonstrated for 4EH (52 g isolated, TON(ethylene) 10.7 · 10^6^) in a 1 L autoclave. Additional to 3MP and 4EH, which are formed with a combined co‐oligomerization selectivity of up to 83 mol‐% in the larger scale experiment, the higher molecular weight co‐oligomers are also interesting, for instance, for plastic production and functionalization chemistry. The authors have cited additional references within the Supporting Information.^[^ ^19^, ^20^, ^21^, ^22^, ^23^ ^]^

## Conflict of Interest

The authors declare no conflict of interest.

## Supporting information

Supporting Information

## Data Availability

The data that support the findings of this study are available in the supplementary material of this article.
