# Supplementary material for: Synthesis of Branched α‐Olefins via Trimerization and Tetramerization of Ethylene
Source: Adv Sci (Weinh). 2024 Aug 9;11(38):2405653. doi: 10.1002/advs.202405653 (PMC11633328; doi:10.1002/advs.202405653)
Supplement: Supplementary file 1 — Supporting Information [file ADVS-11-2405653-s001.pdf]

ADVANCED SCIENCE
Open Access

Supporting Information

for *Adv. Sci.*, DOI 10.1002/advs.202405653

Synthesis of Branched α-Olefins via Trimerization and Tetramerization of Ethylene

*Fabian Lukas, Paula A. Simon, Thomas Dietel, Winfried P. Kretschmer and Rhett Kempe**

Supporting Information for

Synthesis of branched α-olefins via trimerization and tetramerization of ethylene

Fabian Lukas, Paula A. Simon, Thomas Dietel, Winfried P. Kretschmer, Rhett Kempe

Corresponding author: Rhett Kempe, kempe@uni-bayreuth.de

Lehrstuhl Anorganische Chemie II – Catalyst Design, Sustainable Chemistry Centre,

Universität Bayreuth, 95440 Bayreuth, Germany

The file includes:

General methods and materials

Figs. S 1 to S 17

Tables S 1 to S 17

References

General methods and materials

All manipulations were performed with the rigorous exclusion of oxygen and moisture by using standard Schlenk type glassware on a dual-manifold Schlenk line and glovebox techniques (mBraun 120-G) with a high-capacity circulation (< 0.1 ppm O_2) under an atmosphere of argon or nitrogen. Deuterated solvents were obtained from Eurisotop, degassed, distilled and stored over activated 3 Å molecular sieves prior to use. Solvents were dried and purified by distillation from $LiAlH_4$, potassium, Na/K alloy or sodium benzophenone ketyl under argon atmosphere and stored over activated 3 Å molecular sieves before use. 1-Butene (purity: 2.5, Linde AG) was passed over scavenger columns (Supelco Big Moisture Trap Model 23991 and Supelco Big Supelpure^{TM} Model 503088) and ethylene (3.5, Linde AG) over columns of BASF R3-11 supported Cu oxygen scavenger and Al_2O_3 (Fluka). Ethylene pressures are given in bara (*bar absolut*) and ethylene volumes in L_n (22.4 l/mol$_{eth}$ at 273 K). Depleted MAO (d-MAO) was obtained by removing all volatile compounds from a methylalumoxane solution in toluene under reduced pressure. Borate ($[R_2N(CH_3)H]^+[B(C_6F_5)_4]^-$, R = $C_{16}H_{33}$ to $C_{18}H_{37}$) was received from DOW Chemicals. All other reagents and starting materials were purchased from commercial vendors with a purity of at least 97 % and used without further purification unless otherwise noted. 1,3-diphenylimidazolidine-2-imine was prepared according to literature.[1] N-mesityl-6-(2,4,6-triisopropylphenyl)pyridine-2-amidodi(phenylmethanido)chlorido-titanium(IV) (Ap^{9Me}TiBn$_2$Cl), [1,3-bis(2,6-dimethylphenyl)imidazolidin-2-imido]-[N,6-dimesitylpyridin-2-amido-di(phenylmethanido)]-di(phenylmethanido)titanium(IV) (Ap^{6Me}Imi^{Me}TiBn$_2$ (**1**)), [(1,3-bis(2,6-dimethyl-phenyl)imidazolidin-2-ylidene)amido]-[*N*-mesityl-6-(2,4,6-triisopropyl-phenyl)pyridin-2-amido]di(phenylmethanido)titanium(IV) (Ap^{9Me}Imi^{Me}TiBn$_2$ (**2**)) and [1,3-bis(2,6-dimethylphenyl)-imidazolidin-2-imido]-[N-(2,6-diisopropylphenyl)-6-(2,4,6-triisopropylphenyl)-pyridin-2-amido]-di(phenylmethanido)titanium(IV) (Ap*Imi^{Me}TiBn$_2$ (**3**)) were prepared according to our previously published procedure.[2] $Al(OC_6F_5)_3$ was prepared according to literature.[3]

Instruments

Gas chromatography (GC): GC analyses were performed with an Agilent 6890N Network GC system equipped with a HP-5 column (30 m x 320 µm x 0.25 µm) and a flame ionization detector. Gas chromatography / mass spectrometry (GC/MS): GC/MS analyses were carried out on an Agilent 7890B GS system equipped with a HP-5 MS column (30 m x 250 µm x 0.25 µm) and a 5975C inert MSD detector. Standard deviation for 1 mg of substance was determined to be 20 %.

Nuclear magnetic resonance (NMR) spectroscopy: ^1H and ^{13}C NMR spectra were collected on a Varian INOVA 300 (^1H: 300 MHz, ^{13}C: 75 MHz, 20 °C), INOVA 400 (^1H: 399 MHz, ^{13}C: 101 MHz, 20 °C)

spectrometers or a Bruker Avance III HD (^{1}H: 500 MHz, ^{13}C: 125 MHz, 20 °C) spectrometer equipped with a 5 mm CryoProbe^{TM} Prodigy BBO 500 S2 at, chemical shifts (δ) reported in parts per million (ppm) and referenced internally to the residual solvent resonances. Multiplicities are given as follows: s: singlet, d: doublet, t: triplet, q: quartet, quint: quintet, sept: septet, m: multiplet, br: broad signal or combination thereof.

Single crystal structure determination: Suitable crystals for single crystal X-ray diffraction analyses were grown by layering saturated toluene solutions of the complexes (50 mg/0.5 mL) with 0.5 mL of a benzene/hexane mixture (1/5) and cooling to - 27 °C. The X-ray crystal structure analyses were carried out on a STOE-IPDS II or STOE-STADIVARI diffractometer, each equipped with a monochromated molybdenum source (λ(Mo Kα) = 0.71069 Å) and an Oxford Cryostream low-temperature unit (133 K or 180 K). For reflection analysis, integration, determination of unit cell, determination of space group and the numerical absorption correction, X-Area with X-Red32 and LANA were used. Structure solution and refinement was accomplished with OlexSys2[4], SHELXL-2014[5], and Mercury 2020.1[6]. Non-hydrogen atoms were anisotropically refined, hydrogen atoms were included in the refinement on calculated positions riding on their carrier atoms.

Complex synthesis

Synthesis of Lithium[1,3-diphenylimidazolidin-2-ylidene]amide; LiImi^{H}:

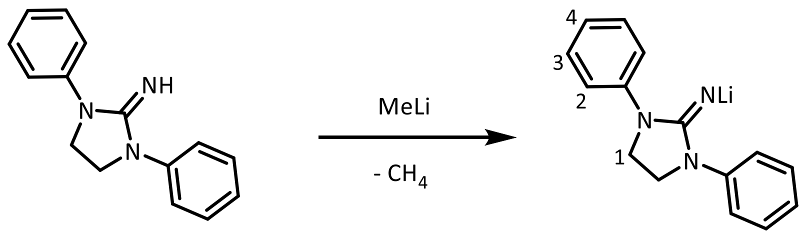

1,3-diphenylimidazolidine-2-imine (1 eq, 4.046 mmol, 960 mg) was dissolved in diethylether (40 mL) and cooled to 0 °C. Upon dropwise addition of MeLi (1.03 eq, 4.167 mmol, 2.6 mL, 1.6 M in diethylether) the reaction mixture was stirred for 30 min at 0 °C and 1 h at room temperature. The product was obtained as a colorless solid after solvent removal.

Yield: 950 mg (96 %)

^{1}H-NMR (399 MHz, C_6D_6, 20 °C): δ = 3.18 (s, 4H, H^{1}), 6.86-6.99 (m, 8H, $H^{2,3}$), 7.27-7.42 (m, 2H, H^{4}) ppm.

Synthesis of [(1,3-diphenylimidazolidin-2-ylidene)amido]-[N-mesityl-6-(2,4,6-triisopropylphenyl)pyridin-2-amido]-di(phenylmethanido)titanium(IV); Ap^{9Me}Imi^{H}TiBn$_2$:

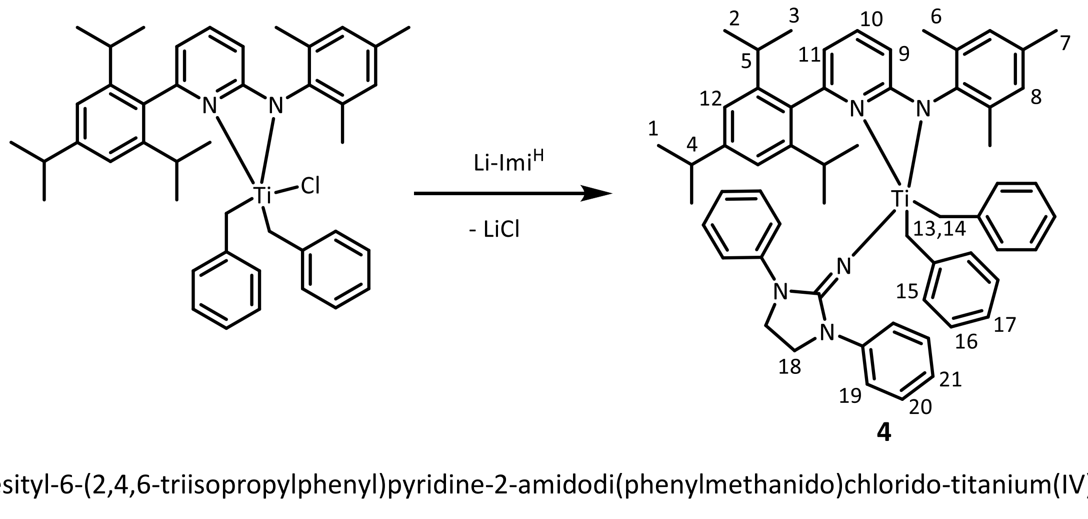

4

N-mesityl-6-(2,4,6-triisopropylphenyl)pyridine-2-amidodi(phenylmethanido)chlorido-titanium(IV) (1.0 eq, 0.294 mmol, 200 mg) and [1,3-diphenylimidazolidin-2-imido]-lithium (1.0 eq, 0.294 mmol, 72 mg) were suspended in hexane/toluene (5/1, 10 mL) und stirred for 2 h at room temperature. Afterwards, the solvent was removed and the residue extracted with hexane (5 mL). The product **4** was obtained as a red solid after solvent removal.

Yield: 110 mg (43 %)

^{1}H-NMR (399 MHz, C_6D_6, 20 °C): δ = 1.25 (d, *J* = 6.7 Hz, 6H, $H^{1,2,3}$), 1.30 (d, *J* = 6.9 Hz, 6H, $H^{1,2,3}$), 1.44 (d, *J* = 6.9 Hz, 6H, $H^{1,2,3}$), 1.59 (s, 6H, H^{6}), 2.20 (s, 3H, H^{7}), 2.70 (d, J = 8.7 Hz, 2H, $H^{13,14}$), 2.80-2.87 (m, 5H, $H^{4,18}$), 2.90 (d, *J* = 8.4 ppm.

^{13}C-NMR (101 MHz, C_6D_6, 20 °C): δ = 18.5 (*C*H_3), 21.0 (*C*H_3), 22.7 (*C*H_3), 24.5 (*C*H_3), 26.9 (*C*H_3), 31.3 (*C*H), 35.0 (C*H*), 44.5 (*C*H_2), 84.8 (*C*H_2), 103.5 (*C*H), 115.2 (*C*H), 121.1 (*C*H), 121.3 (*C*H), 123.1 (*C*H), 124.4 (*C*q), 125.7 (*C*q), 128.8 (*C*H), 129.2 (*C*q), 132.6 (*C*H), 133.1 (*C*H), 135.3 (*C*q), 140.5 (*C*H), 140.7 (*C*q), 140.8 (*C*H), 145.0 (*C*q), 146.8 (*C*q), 147.4 (*C*q), 149.2 (*C*q), 150.0 (*C*q), 156.8 (*C*q), 167.6 (*C*q) ppm.

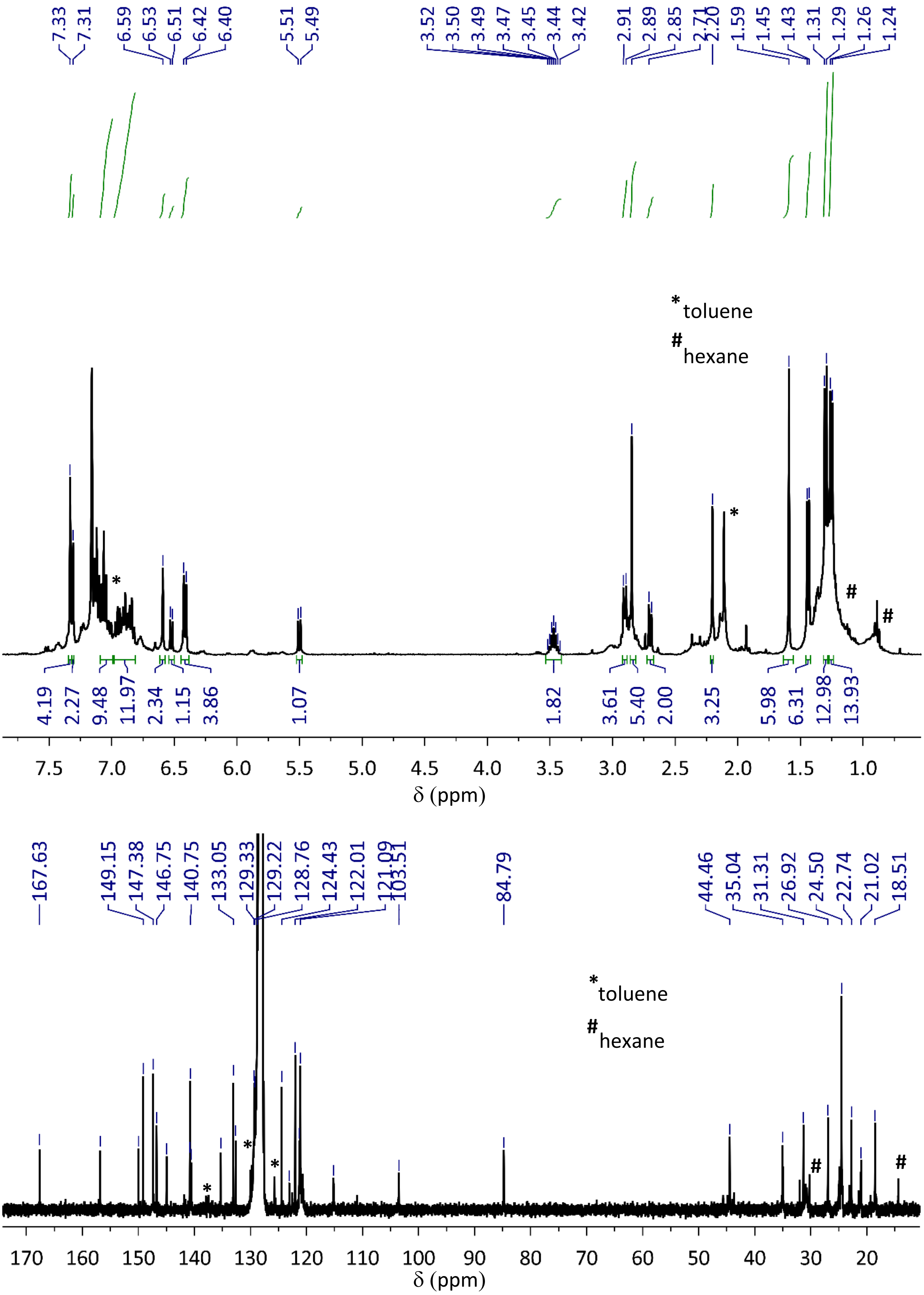

Figure S 1: ^{1}H-NMR spectrum (399 MHz, 20 °C, C_6D_6) and ^{13}C-NMR spectrum (101 MHz, C_6D_6, 20 °C) of Ap^{9Me}Imi^{H}TiBn$_2$.

Crystal structure data

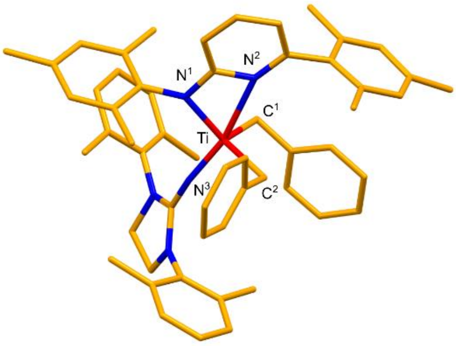

Figure S 2: Molecular structure of Ap^{6Me}Imi^{Me}TiBn$_2$ (1), hydrogen atoms are omitted for clarity. Selected bond lengths (Å) and angles (°). Ti-N^1 2.002(3), Ti-N^2 2.452(3), Ti-N^3 1.808(3), Ti-C^1 2.159(3), Ti-C^2 2.146(3), N^1-Ti-N^2 59.7(1), N^1-Ti-N^3 106.4(1), N^1-Ti-C^1 117.4(1), C^1-Ti-C^2 112.1(1).

Table S 1: Data of the crystal structure determination for 1.

Empirical formula	$C_{62}H_{67}N_5Ti$
Formula weight	930.10
Temperature	133 K
Wavelength	MoKα (λ = 0.71069)
Crystal system	orthorhombic
Space group	Fdd2
Unit cell dimensions	a = 22.794(5) Å; α = 90.000(5) °
	b = 56.813(5) Å; β = 90.000(5) °
	c = 16.283(5) Å; γ = 90.000(5) °
Volume	21086(8) Å^3
Z	16
Density (calculated)	1.172 g/cm^3
Absorption coefficient	0.206 mm^{-1}
F(000)	7936.0
Crystal size	0.565 x 0.427 x 0.401 mm^3
2θ range for data collection	2.868 to 51.998 °
Index ranges	-28 ≤ h ≤ 26, -70 ≤ k ≤ 44, -16 ≤ l ≤ 20
Reflections used	19302
Independent reflections	7816 [R_{int} = 0.0499, R_{sigma} = 0.1099]
Completeness	98.8 %
Absorption correction	numerical
Min. and max. transmission	0.9784 and 0.9877
Goodness-of-fit	0.716
Final *R* indices [*I*>2sigma(*I*)]	R_1 = 0.0381, wR_2 = 0.0580
R indices (all data)	R_1 = 0.0688, wR_2 = 0.0644
Largest diff. Peak and deepest hole	0.17/-0.25 e Å^{-3}

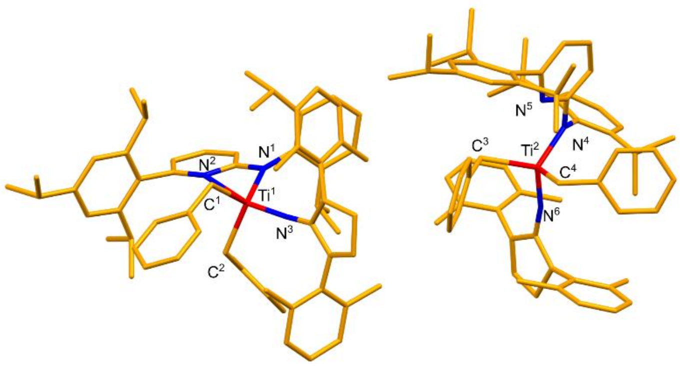

Figure S 3: Molecular structure of Ap*Imi^{Me}TiBn$_2$ (3), hydrogen atoms are omitted for clarity. Disordered solvent (benzene) was squeezed out. Selected bond lengths (Å) and angles (°). Ti^1-N^1 2.021(3), Ti^1-N^2 2.534(3), Ti^1-N^3 1.806(3), Ti^1-C^1 2.151(3), Ti^1-C^2 2.129(3), N^1-Ti^1-N^2 57.9(1), N^1-Ti^1-N^3 106.2(1), N^1-Ti^1-C^1 120.1(1), C^1-Ti^1-C^2 106.2(1); Ti^2-N^4 1.979(3), Ti^2-N^6 1.816(3), Ti^2-C^3 2.136(3), Ti^2-C^4 2.137(3), N^4-Ti^2-N^6 110.7(1), N^4-Ti^2-C^3 113.2(1), C^3-Ti^2-C^4 113.0(1).

Table S 2: Data of the crystal structure determination for 3.

Empirical formula	$C_{136}H_{164}N_{10}Ti_2$
Formula weight	2034.56
Temperature	180 K
Wavelength	MoKα (λ = 0.71073)
Crystal system	triclinic
Space group	P-1
Unit cell dimensions	a = 17.250(3) Å; α = 107.20(3) °
	b = 18.640(4) Å; β = 112.40(3) °
	c = 21.050(4) Å; γ = 91.00(3) °
Volume	5913(3) Å^3
Z	2
Density (calculated)	1.143 g/cm^3
Absorption coefficient	0.189 mm^{-1}
F(000)	2188.0
Crystal size	0.391 x 0.241 x 0.176 mm^3
2θ range for data collection	2.312 to 57.138 °
Index ranges	-22 ≤ h ≤ 14, -24 ≤ k ≤ 24, -25 ≤ l ≤ 28
Reflections used	81758
Independent reflections	28123 [R_{int} = 0.1124, R_{sigma} = 0.1962]
Completeness	93.2 %
Absorption correction	numerical
Min. and max. transmission	0.992 and 0.962
Goodness-of-fit	0.809
Final *R* indices [*I*>2sigma(*I*)]	R_1 = 0.0642, wR_2 = 0.1260
R indices (all data)	R_1 = 0.1629, wR_2 = 0.1560
Largest diff. Peak and deepest hole	0.59/-0.37 e Å^{-3}

η^2–Ap^{6Me} Imi^{Me} Ti Bn$_2$ η^2–Ap* Imi^{Me} Ti Bn$_2$

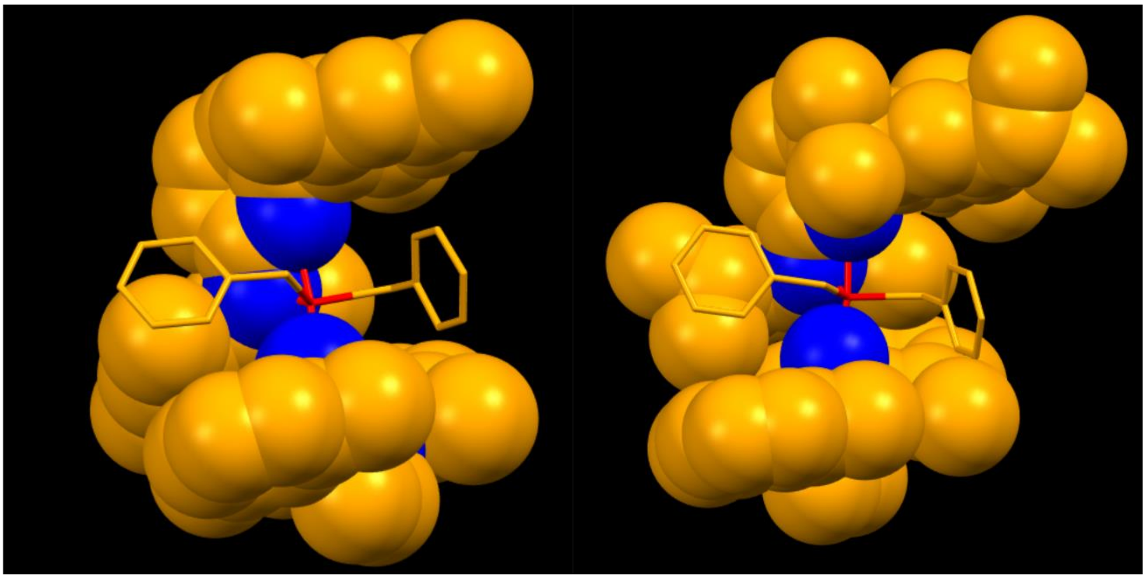

Figure S 4: Spacefill visualisation of η^2-Ap^{6Me}Imi^{Me}TiBn$_2$ and Ap*Imi^{Me}TiBn$_2$ to demonstrate the different sterics at the titanium center in the solid state. The benzyl ligands are displayed as capped sticks for clarity.

General procedure for ethylene oligomerization

All ethylene oligomerization reactions were carried out in a Büchi 50 mL glass autoclave equipped with a Bronkhorst HIGH-TECH El-FLOW meter, a mechanical stirrer, injection system and a 10 bar over pressure valve. The injection system was secured with a rubber septum to avoid loss of pressure during injection. The reactors were temperature and pressure controlled and the runs were conducted in semi batch mode (ethylene was added by replenishing flow to keep the pressure constant). Prior to use, the reactor was evacuated and heated for 30 min at 50 °C, charged with 18.5 mL methylcyclohexane, brought to the desired temperature and stirred at 1000 rpm. After pressurizing with ethylene at the desired pressure, the autoclave was equilibrated for 5 minutes. Subsequently, 0.5 mL TIBA scavenger (30 µmol, 0.06 M stock solution), 0.5 mL activator solution (110 nmol borate, 0.22 mM stock solution in methylcyclohexane; for precatalyst $Ap^{9Me}Imi^{H}TiBn_2$: 1.1 µmol borate, 2.2 mM stock solution) and 0.5 mL precatalyst solution (100 nmol, 0.2 mM stock solution in methylcyclohexane; for precatalyst $Ap^{9Me}Imi^{H}TiBn_2$: 1.0 µmol, 2 mM stock solution) were injected. The ethylene pressure was kept constant during the run to within 0.1 bar of the initial pressure by replenishing the gas flow. After the desired ethylene volume was consumed, the ethylene flow stopped, 1 mL diluted HCl_{aq} added, and the reactor cooled to 0 °C (Attention! 1-butene is a liquid gas! Bp = −6.26 °C at 1 bara, do not exceed 75 °C when using a glass autoclave!). The pressure was released, internal standard added, and the solution analyzed by GC to determine the product distribution. The activity was calculated based on the ethylene flow.

General procedure for 1-butene/ethylene co-oligomerization

All 1-butene/ethylene co-oligomerization reactions were carried out in a Büchi 50 mL glass autoclave equipped with a Bronkhorst HIGH-TECH El-FLOW meter, a mechanical stirrer, injection systems and a 10 bar over pressure valve. Large-scale reactions were carried out in a Büchi 1 L stainless steel autoclave. The injection systems were secured with rubber septa to avoid loss of 1-butene during injection. The reactors were temperature and pressure controlled and the runs were conducted in semi batch mode (ethylene was added by replenishing flow to keep the pressure constant). Prior to use, the reactor was evacuated and heated for 30 min at 50 °C, charged with 1-butene via condensation and additional solvent (for 50 mmol 1-butene runs: V_{SUM} = 15 mL; for large-scale reactions: no additional solvent), brought to the desired temperature and stirred at 1000 rpm. After pressurizing with ethylene at the desired pressure the autoclave was equilibrated for 5 minutes. Subsequently, 0.5 mL TIBA scavenger (for 50 mmol 1-butene runs: 30 µmol, 0.06 M stock solution; for large-scale reactions: 200 µmol, 0.4 M stock solution), 0.5 mL activator solution (for 50 mmol 1-butene runs: 110 nmol borate, 0.22 mM stock solution in methylcyclohexane; for large-scale reactions: 75 µmol d-MAO, 0.15 M, stock solution in cumene) and 0.5 mL precatalyst solution (for 50 mmol 1-

butene runs: 100 nmol, 0.2 mM stock solution in methylcyclohexane; for large-scale reactions: 500 nmol, 1.0 mM stock solution in methylcyclohexane) were injected. The ethylene pressure was kept constant during the run to within 0.1 bar of the initial pressure by replenishing the gas flow. After the desired ethylene volume was consumed, the ethylene flow was stopped, 1 mL diluted HCl_{aq} added, and the reactor cooled to 0 °C (Attention! 1-butene is a liquid gas! Bp = −6.26 °C at 1 bara, do not exceed 75 °C when using a glass autoclave!). The pressure was released, internal standard added, and the solution analyzed by GC to determine the product distribution. The activity was calculated based on the ethylene flow.

General procedure for ethylene tri-/tetramerization

All ethylene tri-/tetramerization reactions were carried out in a Büchi 50 mL glass autoclave equipped with a Bronkhorst HIGH-TECH El-FLOW meter, a mechanical stirrer, injection systems and a 10 bar over pressure valve. Prior to use, the reactor was evacuated and heated for 30 min at 50 °C, brought to the desired temperature, charged with 8 mL solvent, and stirred at 1000 rpm. After pressurizing with ethylene at the desired pressure the autoclave was equilibrated for 5 minutes. Subsequently, 0.5 mL TIBA scavenger (30 µmol, 0.06 M stock solution), 0.5 mL activator solution (For borate and $Al(OPh^F)_3$: 330 nmol, 0.6 mM stock solution; for d-MAO: 75 µmol, 0.15 M, stock solution) and 0.5 mL precatalyst solution (300 nmol, 0.6 mM stock solution) were injected. The ethylene pressure was kept constant during the run to within 0.1 bar of the initial pressure by replenishing the gas flow. After the desired ethylene volume was consumed, the ethylene flow was stopped, 1 mL diluted HCl_{aq} was added, and the reactor was cooled to 0 °C (Attention! 1-butene is a liquid gas! bp = −6.26 °C at 1 bara, do not exceed 75 °C when using a glass autoclave!). The pressure was released, internal standard added, and the solution analyzed by GC to determine the product distribution. The activity was calculated based on the ethylene flow.

Results of the ethylene oligomerization

Major co-oligomer products (1-butene + ethylene)

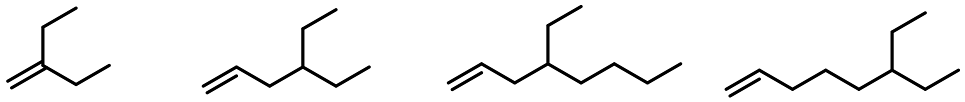

3-methylenepentane 3MP | 4-ethylhex-1-ene 4EH | 4-ethyloct-1-ene 4EO | 6-ethyloct-1-ene 6EO

Major co-oligomer products (1-hexene + ethylene)

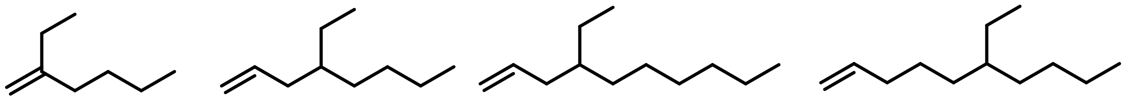

3-methyleneheptane 3MH | 4EO | 4-ethyldec-1-ene 4ED | 6-ethyldec-1-ene 6ED

Precatalysts

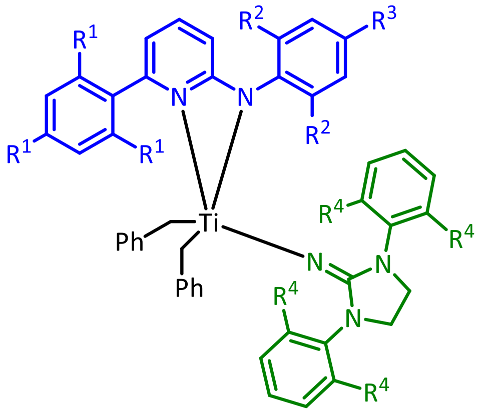

Ap^{6Me}: $R^1 = R^2 = R^3$ = Me

Ap^{9Me}: R^1 = ^{i}Pr; $R^2 = R^3$ = Me

Ap*: R^1 = ^{i}Pr; R^2 = ^{i}Pr; R^3 = H

Imi^H: R^4 = H

Imi^{Me}: R^4 = Me

Precatalyst	Ap	Imi
**1**	6Me	Me
**2**	9Me	Me
**3**	*	Me
**4**	9Me	H

Activators

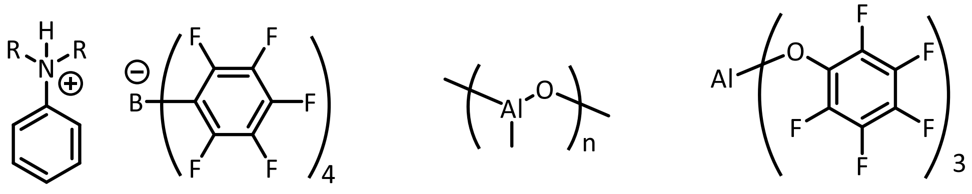

R: $C_{16}H_{33}$ to $C_{18}H_{37}$

borate | d-MAO | Al(Ph^F)$_3$

Figure S 5: Main co-oligomer products of the ethylene oligomerization (top) using catalyst systems consisting of precatalysts 1 – 4 (middle) and activators (bottom).

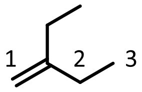
1
2
3

3-methylenepentane (C_6H_{12}, M = 84.16 g/mol): ^{1}H-NMR (399 MHz, C_6D_6, 20 °C): δ = 0.97 (t, *J* = 7.5 Hz, 6H, H^3), 1.94 (qt, *J* = 7.5, 1.2 Hz, 4H, H^2), 4.81 (quint, *J* = 1.2 Hz, 2H, H^1) ppm. ^{13}C-NMR (101 MHz, C_6D_6, 20 °C): δ = 12.64, 29.22, 106.90, 152.91 ppm.

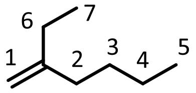
6
7
1
2
3
4
5

3-methyleneheptane (C_8H_{16}, M = 112.22 g/mol): ^{1}H-NMR (399 MHz, $CDCl_3$, 20 °C): δ = 0.91 (t, *J* = 7.5 t, *J* = 7.5 δ = 12.54, 14.16, 22.66, 28.88, 30.23, 36.12, 107.39, 152.07 ppm.

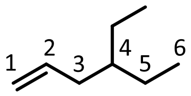
1
2
3
4
5
6

4-ethylhex-1-ene (C_8H_{16}, M = 112.22 g/mol): ^{1}H-NMR (500 MHz, $CDCl_3$, 20 °C): δ = 0.85 (t, *J* = 7.2 Hz, 6H, H^6), 1.21 – 1.32 (m, 5H, $H^{4,5}$), 2.00 – 2.04 (m, 2H, H^3), 4.95 – 5.02 (m, 2H, H^2), 5.77 (ddt, J = 17.2, 10.1, 7.2 Hz, 1H H^1) ppm. ^{13}C-NMR (125 MHz, $CDCl_3$, 20 °C): δ = 11.12, 25.41, 37.40, 40.61, 115.51, 137.91 ppm.

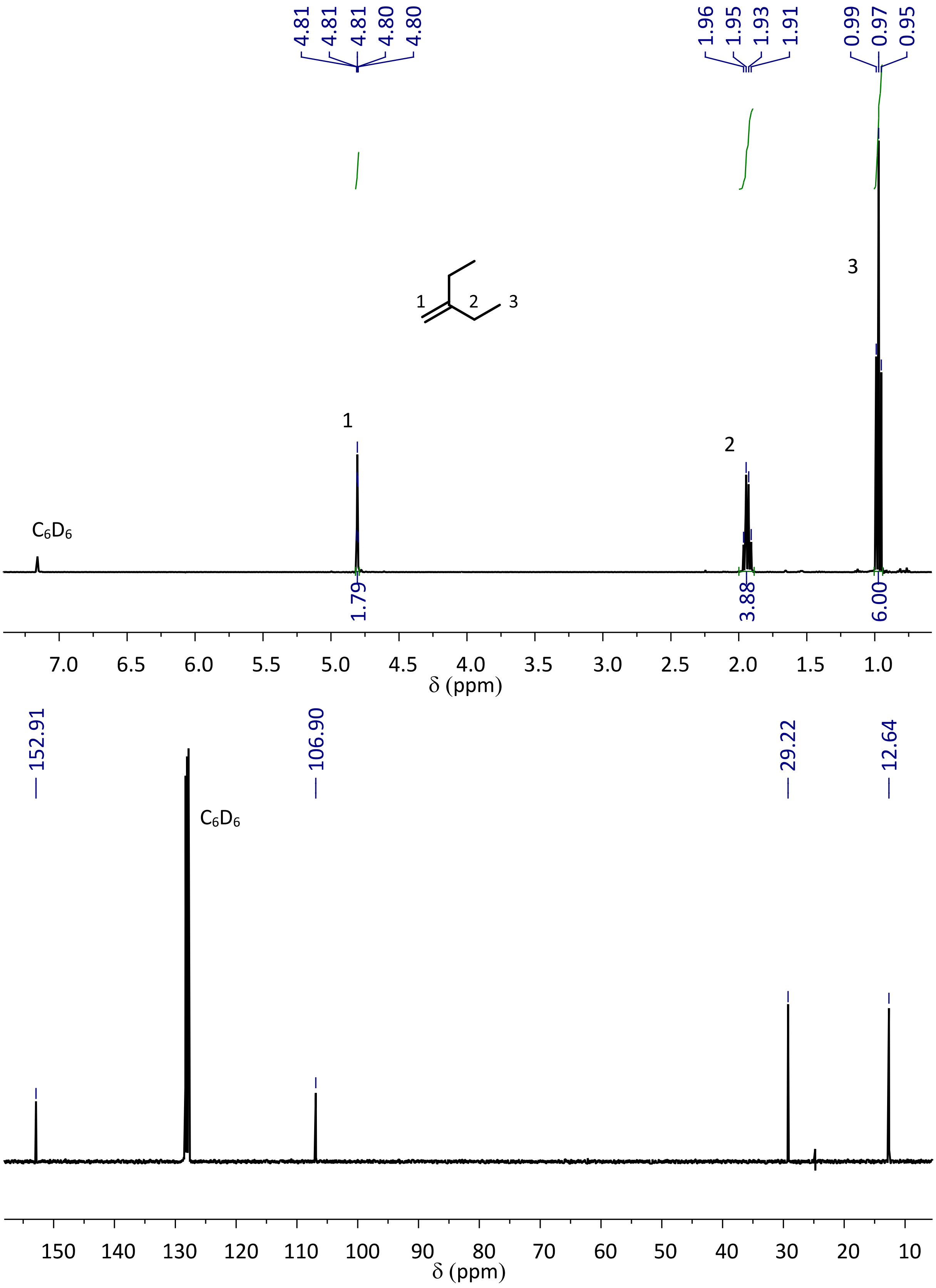

Figure S 6: ^{1}H-NMR spectrum (399 MHz, 20 °C, C_6D_6) and ^{13}C-NMR spectrum (101 MHz, C_6D_6, 20 °C) of 3MP.

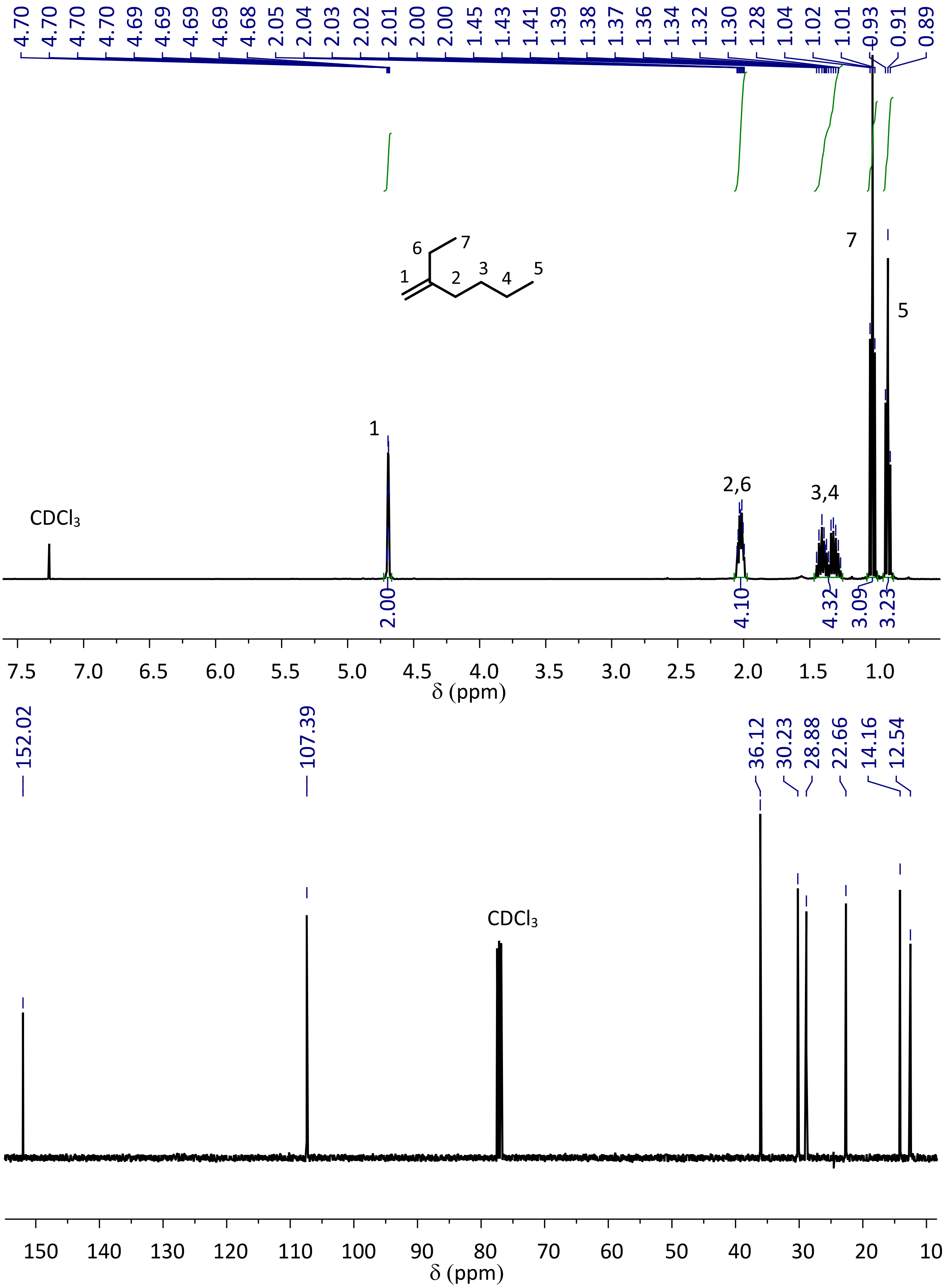

Figure S 7: ^{1}H-NMR spectrum (399 MHz, 20 °C, $CDCl_3$) and ^{13}C-NMR spectrum (101 MHz, $CDCl_3$, 20 °C) of 3MH.

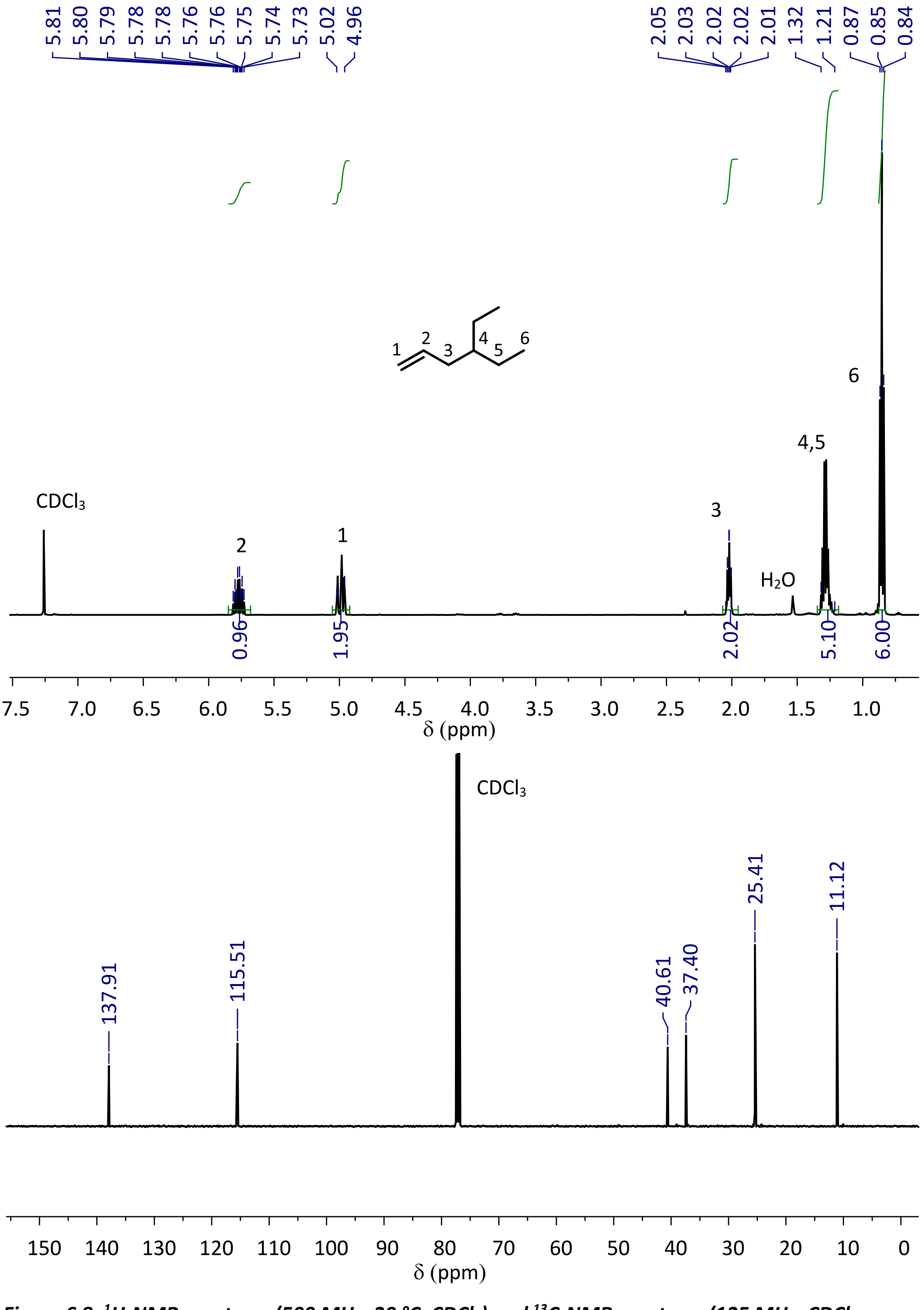

Figure S 8: ^{1}H-NMR spectrum (500 MHz, 20 °C, $CDCl_3$) and ^{13}C-NMR spectrum (125 MHz, $CDCl_3$, 20 °C) of 4EH.

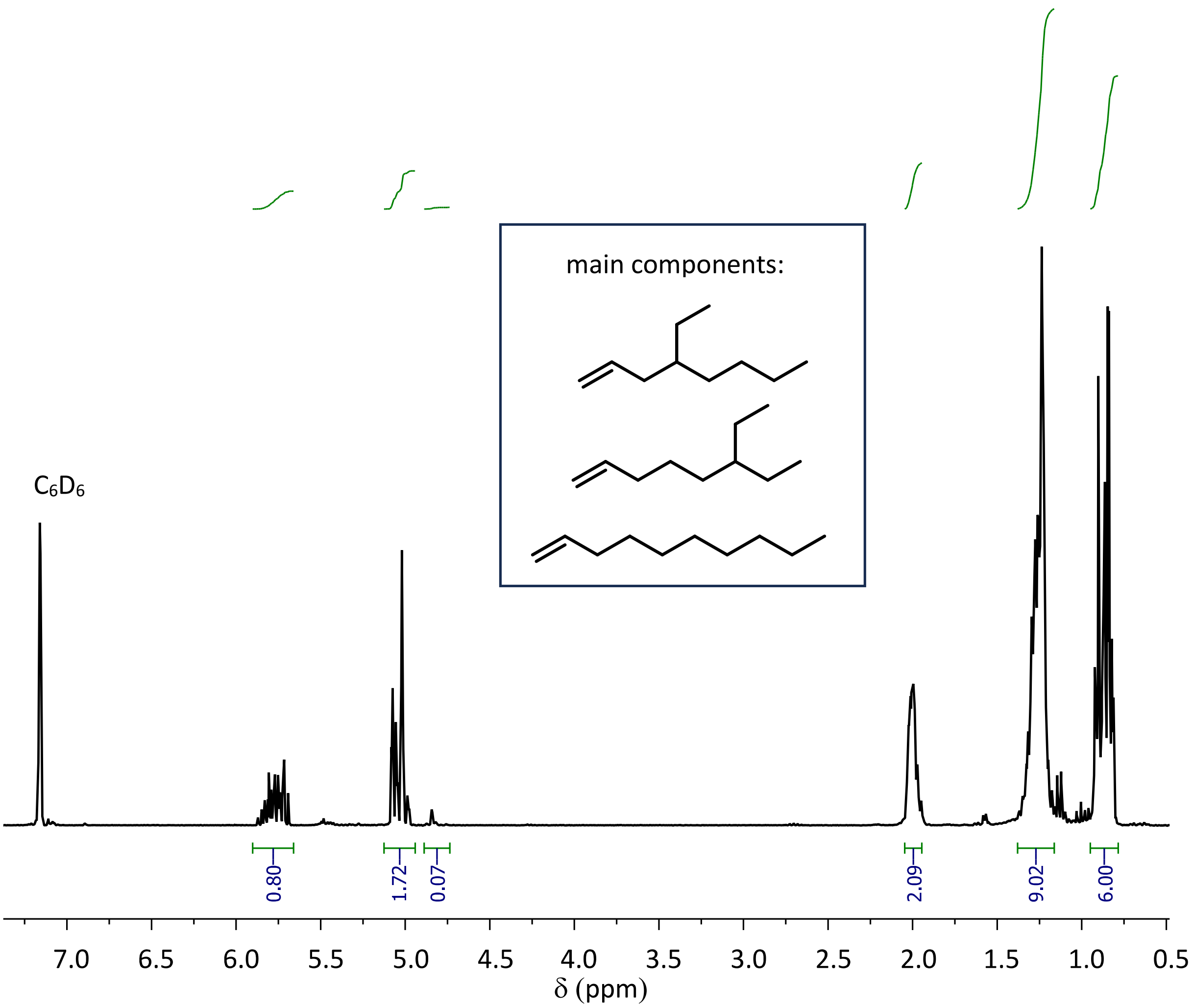

Figure S 9: ^{1}H-NMR spectrum (300 MHz, 20 °C, C_6D_6) of the C10-fraction after distillation which was produced using 3 in the ethylene tetramerization (table S 10, entry 29). The main components include 1-decene, 4EO and 6EO.

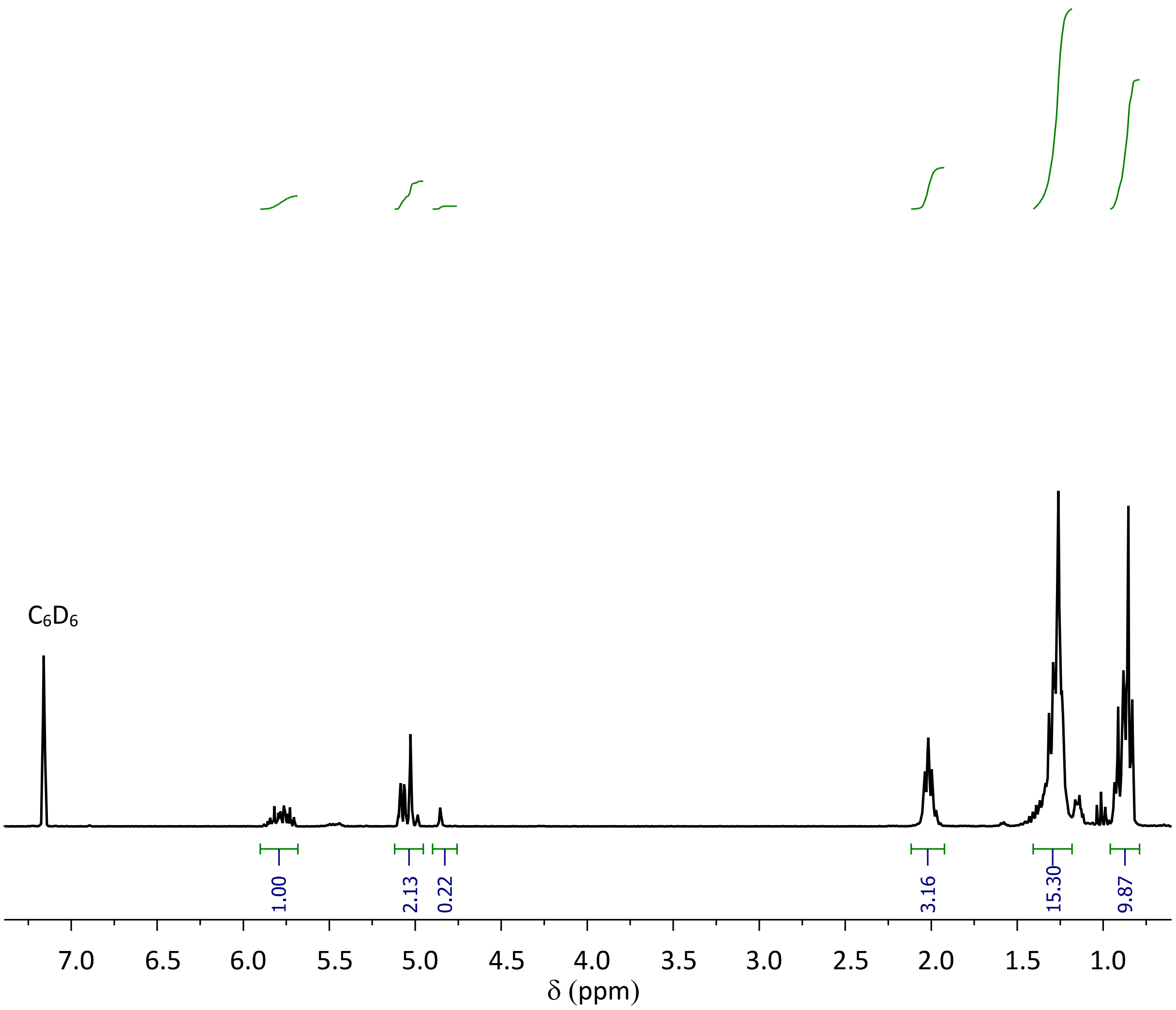

Figure S 10: ^{1}H-NMR spectrum (300 MHz, 20 °C, C_6D_6) of the C12-fraction after distillation which was produced using 3 in the ethylene tetramerization (table S 10, entry 29).

Table S 3: Influence of the precatalyst (1 - 4) on the ethylene oligomerization.

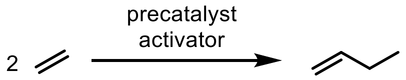

**Entry**	precatalyst	α	x(linear) / mol-%	Activity / $kg_{Eth}\ mol_{Ti}^{-1}\ h^{-1}\ bar^{-1}$
**1**	1	0.11	99	204 000
**2**	2	0.10	97	364 900
**3**	3	0.20	98	147 200
**4**	4	0.33	99	7 000

Reaction conditions: n(precatalyst) = 100 nmol; activator: borate; $\frac{n(borate)}{n(Ti)}$ = 1.1; T = 20 °C; p = 1.7 bar; V(eth) = 0.25 L_n; n(TIBA) = 30 µmol; V(sum) = 20 mL; solvent: methylcyclohexane; scavenger: triisobutylaluminum (TIBA); internal standard: cumene.

Table S 4: Overall mass and wt-% of the ethylene oligomerization runs (precatalyst comparison study; table S 1).

**Entry**	m(1But) / mg (wt-%)	m(1Hex) / mg (wt-%)	m(1Oct) / mg (wt-%)	m(1Dec) / mg (wt-%)	m(co-oligomers) / mg (wt-%)
**1**	257 (76)	42 (13)	6 (2)	1 (<1)	6 (2)
**2**	257 (82)	39 (12)	5 (2)	1 (<1)	11 (4)
**3**	215 (69)	65 (21)	17 (5)	5 (2)	12 (4)
**4**	160 (51)	79 (25)	35 (11)	24 (7)	15 (5)

No polymer formation was observed. 1-Butene content was calculated using the α-value.

Table S 5: Influence of the precatalyst (1 - 3) on the co-oligomerization of 1-butene and ethylene.

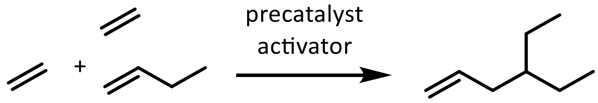

Entry	Precatalyst ApImiTiBn$_2$	Co-oligomers / mol-%					α	Activity / kg_{Eth} mol_{Ti}^{-1} h^{-1} bar^{-1}
		3MP	4EH	4EO	6EO	C12		
**5**	1	46	39	7	2	3	0.12	9 300
**6**	2	38	46	5	4	5	0.11	14 300
**7**	3	10	69	5	8	7	0.20	11 800

Reaction conditions: n(Ti) = 100 nmol; activator: borate; $\frac{n(borate)}{n(Ti)}$ = 1.1; scavenger: n(TIBA) = 30 µmol; T = 20 °C; p(eth) = 1.7 bar; V(eth) = 1 L_n; n(1-butene) = 50 mmol; solvent: methylcyclohexane; V(sum) = 15 mL; internal standard: cumene or methylcyclohexane; selectivity based on hetero-oligomer distribution.

Table S 6: Overall mass and wt-% of the co-trimerization of 1-butene and ethylene using precatalyst 1 - 3 (table S 5).

Entry	m(1But) / mg (wt-%)	m(1Hex) / mg (wt-%)	m(1Oct) / mg (wt-%)	m(3MP) / mg (wt-%)	m(4EH) / mg (wt-%)	m(3MH) / mg (wt-%)	m(4EO) / mg (wt-%)	m(6EO) / mg (wt-%)	m(C12) /mg (wt-%)
**5**	671 (39)	121 (7)	40 (2)	323 (19)	368 (22)	30 (2)	85 (5)	26 (2)	42 (2)
**6**	471 (24)	78 (4)	11 (1)	419 (21)	665 (34)	29 (1)	90 (5)	72 (4)	73 (4)
**7**	550 (28)	165 (8)	47 (2)	65 (3)	591 (30)	< 1 (< 1)	55 (3)	87 (4)	76 (4)

No polymer formation was observed. 1-Butene content was calculated using the α-value.

Table S 7: Co-oligomerization of 1-butene and ethylene using $Ti(OBu)_4/AlEt_3$ (Alphabutol type catalyst).

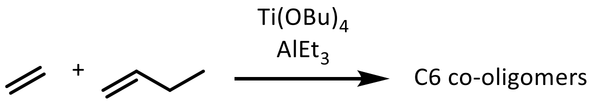

**Entry**	Co-oligomers / mol-%				Activity / $kg_{Eth}\ mol_{Ti}^{-1}\ h^{-1}\ bar^{-1}$
	3MP	3-methylpent-1-ene	3-methylpentane	C8	
**8**	57	29	5	9	4

Reaction conditions: $n(Ti(OBu)_4)$ = 300 µmol; activator: $AlEt_3$; $\frac{n(Al)}{n(Ti)} = 3$; T = 20 °C; p(eth) = 1.7 bar; V(eth) = 1 L_n; n(1-butene) = 50 mmol; solvent: methylcyclohexane; V(sum) = 15 mL; internal standard: cumene; selectivity based on hetero-oligomer distribution.

Table S 8: Overall mass and wt-% of the co-oligomerization of 1-butene and ethylene using $Ti(OBu)_4/AlEt_3$ (table S 7).

**Entry**	m(1But) / mg (wt-%)	m(1Hex) / mg (wt-%)	m(3MP) / mg (wt-%)	m(3Me1Pentene) / mg (wt-%)	m(3MePentane) / mg (wt-%)	m(C8) / mg (wt-%)	m(Polymer) / mg (wt-%)
**8**	1130 (78)	14 (1)	158 (11)	80 (6)	15 (1)	33 (2)	5 (< 1)

Table S 9: Ethylene solubility in methylcyclohexane as the solvent at various temperatures and ethylene pressures. The solubilities were determined via gravimetric measurements with different 1-butene volumes, ethylene pressures and temperatures.

T (°C)	p (bara)	c(ethylene) ($\frac{\mu mol}{mL}$)
-10	1.5	376 ± 10
	2.0	457 ± 13
	2.5	601 ± 16
0	1.5	289 ± 5
	2.0	352 ± 10
	2.5	435 ± 7
10	1.5	241 ± 5
	2.0	319 ± 14
	2.5	376 ± 7
20	1.5	219 ± 7
	2.0	266 ± 5
	2.5	303 ± 7

Table S 10: Influence of temperature, ethylene pressure, ethylene volume, solvent, and activator on the ethylene trimerization using precatalyst 1.

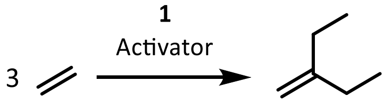

Entry	Act. (solv.)	V / L_n	T / °C (p / bar)	Co-oligomers / mol-%					α	Activity / kg_{Eth} mol_{Ti}^{-1} h^{-1} bar^{-1}
				3MP	3MH	4EH	4EO+6EO	C12		
**9**	d-MAO (MeCy)	1	20 (1.7)	35	4	36	18	5	0.12	35 600
**10**	$Al(OPh^F)_3$ (MeCy)	1	20 (1.7)	38	3	41	13	3	0.10	17 200
**11**	borate (MeCy)	1	20 (1.7)	40	4	33	16	6	0.13	40 100
**12**	borate (cumene)	1	20 (1.7)	35	4	34	19	6	0.14	57 500
**13**	borate (C_6H_5Cl)	1	20 (1.7)	36	3	37	17	5	0.12	56 000
**14**	borate (MeCy)	1	20 (3.0)	29	3	37	22	7	0.17	68 300
**15**	borate (MeCy)	1	- 10 (0.9)	58	4	25	10	2	0.08	50 000
**16**	borate (MeCy)	1	0 (1.2)	55	3	28	11	2	0.09	62 700
**17**	d-MAO (MeCy)	1	0 (1.2)	50	3	35	10	2	0.08	55 100
**18**	borate (MeCy)	1	0 (3.0)	35	3	36	20	5	0.15	144 000
**19**	borate (MeCy)	0.5	0 (1.2)	54	1	29	11	5	0.10	65 900
**20**	borate (MeCy)	3	0 (1.2)	50	4	28	12	5	0.11	11 900

Reaction conditions: n(Ti) = 300 nmol; activator: d-MAO, borate or $Al(OPh^F)_3$; $\frac{n(d\text{-}MAO)}{n(Ti)} = 250$; $\frac{n(borate)}{n(Ti)} = \frac{n(Al(OPhF)3)}{n(Ti)} = 1.1$ scavenger: n(TIBA) = 30 µmol; V(solvent) = 10 mL; internal standard: cumene or methylcyclohexane; selectivity based on hetero-oligomer distribution.

Table S 11: Overall mass and wt-% of the ethylene trimerization (table S 10).

**Entry**	m(1But) / mg (wt-%)	m(1Hex) / mg (wt-%)	m(1Oct) / mg (wt-%)	m(3MP) / mg (wt-%)	m(4EH) / mg (wt-%)	m(3MH) / mg (wt-%)	m(4EO) / mg (wt-%)	m(6EO) / mg (wt-%)	m(C12) /mg (wt-%)
**9**	936 (75)	168 (13)	27 (2)	31 (2)	42 (3)	4 (< 1)	24 (2)	3 (< 1)	9 (1)
**10**	992 (79)	149 (12)	21 (2)	27 (2)	38 (3)	3 (< 1)	14 (1)	2 (< 1)	4 (< 1)
**11**	941 (75)	183 (14)	32 (3)	27 (2)	29 (2)	3 (< 1)	15 (1)	2 (< 1)	6 (< 1)
**12**	885 (71)	186 (15)	37 (3)	34 (3)	44 (4)	5 (< 1)	27 (2)	4 (< 1)	11 (1)
**13**	919 (73)	165 (13)	28 (2)	36 (3)	50 (4)	4 (< 1)	26 (2)	4 (< 1)	10 (1)
**14**	869 (70)	222 (18)	50 (4)	21 (2)	36 (3)	3 (< 1)	23 (2)	4 (< 1)	11 (1)
**15**	973 (78)	117 (9)	13 (1)	72 (6)	42 (3)	7 (1)	19 (2)	2 (< 1)	6 (< 1)
**16**	958 (77)	129 (10)	16 (1)	66 (5)	45 (4)	5 (< 1)	20 (2)	1 (< 1)	6 (< 1)
**17**	978 (78)	117 (9)	11 (1)	58 (5)	56 (5)	4 (< 1)	15 (1)	4 (< 1)	5 (< 1)
**18**	910 (73)	205 (16)	41 (3)	23 (2)	32 (3)	3 (< 1)	19 (2)	3 (< 1)	7 (1)
**19**	491 (79)	74 (12)	10 (2)	22 (4)	16 (3)	2 (< 1)	7 (1)	1 (< 1)	2 (< 1)
**20**	2318 (62)	382 (10)	55 (1)	391 (10)	290 (8)	50 (1)	139 (4)	18 (< 1)	73 (2)

No polymer formation was observed. 1-Butene content was calculated using the α-value.

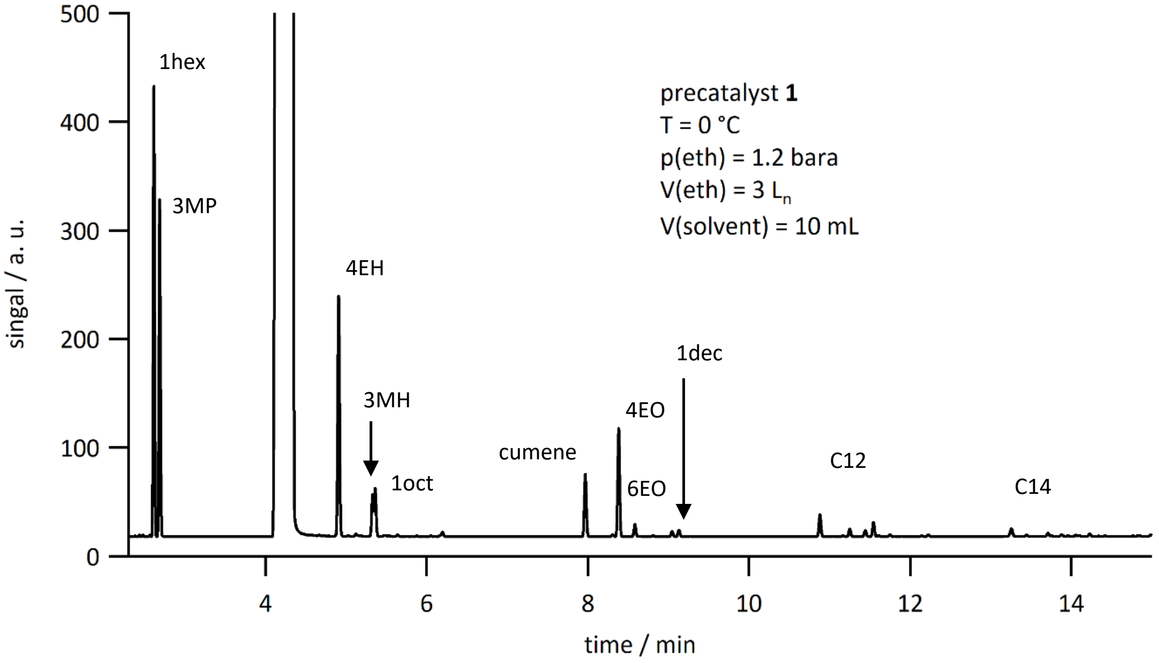

Figure S 11: Gas chromatogram of the products obtained from the ethylene trimerization run at 0 °C and 1.2 bara pressure using precatalyst 1 (Table S10, entry 20, 3 L_n ethylene).

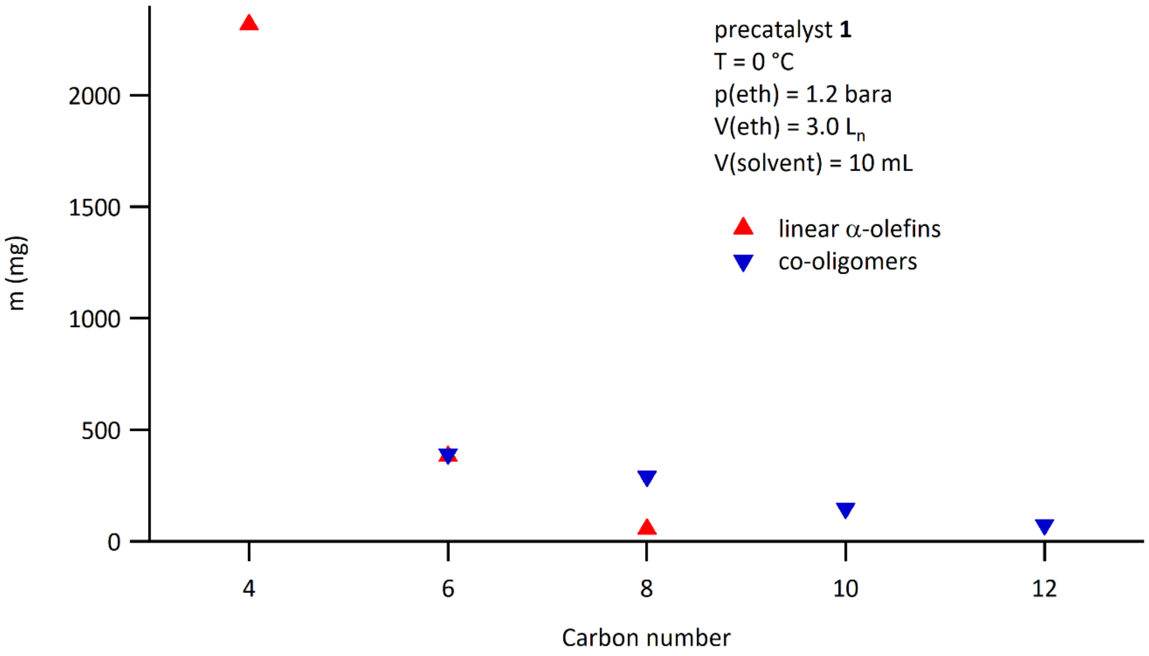

Figure S 12: Plot of detected olefin masses (GC) against the carbon number from ethylene trimerization (table S 10, entry 20).

Table S 12: Influence of temperature, ethylene pressure, ethylene volume, solvent, and activator on the ethylene tetramerization using precatalyst 3.

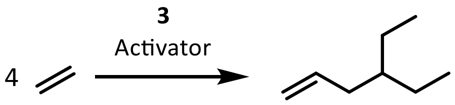

Entry	Act. (solv.)	V / L_n	T / °C (p / bar)	Co-oligomers / mol-%					α	Activity / kg_{Eth} mol_{Ti}^{-1} h^{-1} bar^{-1}
				3MP	3MH	4EH	4EO+6EO	C12		
**21**	d-MAO (MeCy)	1	20 (1.7)	7	< 1	59	26	9	0.20	39 700
**22**	$Al(OPh^F)_3$ (MeCy)	1	20 (1.7)	/	/	/	/	/	/	/
**23**	borate (MeCy)	1	20 (1.7)	9	< 1	55	25	8	0.21	40 700
**24**	d-MAO (cumene)	1	20 (1.7)	7	< 1	55	28	8	0.22	27 800
**25**	d-MAO (C_6H_5Cl)	1	20 (1.7)	8	< 1	56	25	8	0.19	22 500
**26**	d-MAO (MeCy)	1	20 (3.0)	5	< 1	48	29	13	0.25	32 000
**27**	d-MAO (MeCy)	1	- 10 (0.9)	13	< 1	66	17	3	0.12	25 400
**28**	d-MAO (MeCy)	1	0 (1.2)	10	< 1	64	20	5	0.15	7 000
**29**	RIBS (MeCy)	1	0 (1.2)	13	< 1	58	20	6	0.16	10 200
**30**	d-MAO (MeCy)	1	0 (3.0)	8	< 1	57	25	8	0.19	39 300
**31**	d-MAO (MeCy)	0.5	0 (1.2)	11	< 1	67	18	4	0.15	111 900
**32**	d-MAO (MeCy)	3	0 (1.2)	10	< 1	62	19	6	0.15	4 900

Reaction conditions: n(Ti) = 300 nmol; activator: d-MAO, borate or $Al(OPh^F)_3$; $\frac{n(\text{d-MAO})}{n(Ti)}$ = 250; $\frac{n(\text{borate})}{n(Ti)}$ = $\frac{n(Al(OPhF)3)}{n(Ti)}$ = 1.1 scavenger: n(TIBA) = 30 µmol; V(solvent) = 10 mL; internal standard: cumene or methylcyclohexane; selectivity based on hetero-oligomer distribution.

Table S 13: Overall mass and wt-% of the ethylene tetramerization (table S 12).

**Entry**	m(1But) / mg (wt-%)	m(1Hex) / mg (wt-%)	m(1Oct) / mg (wt-%)	m(3MP) / mg (wt-%)	m(4EH) / mg (wt-%)	m(3MH) / mg (wt-%)	m(4EO) / mg (wt-%)	m(6EO) / mg (wt-%)	m(C12) /mg (wt-%)
**21**	779 (62)	245 (20)	64 (5)	7 (1)	79 (6)	< 1 (< 1)	35 (3)	10 (1)	12 (1)
**22**	/	/	/	/	/	/	/	/	/
**23**	809 (65)	255 (20)	70 (6)	6 (< 1)	48 (4)	< 1 (< 1)	20 (2)	8 (1)	11 (1)
**24**	770 (62)	242 (19)	69 (6)	7 (1)	75 (6)	< 1 (< 1)	34 (3)	12 (1)	17 (1)
**25**	810 (65)	231 (18)	58 (5)	7 (1)	71 (6)	< 1 (< 1)	30 (2)	9 (1)	15 (1)
**26**	713 (57)	267 (21)	90 (7)	5 (< 1)	62 (5)	< 1 (< 1)	34 (3)	13 (1)	24 (2)
**27**	913 (73)	164 (13)	25 (2)	13 (1)	91 (7)	11 (1)	24 (2)	6 (< 1)	7 (< 1)
**28**	845 (68)	190 (15)	37 (3)	12 (1)	103 (8)	< 1 (< 1)	33 (3)	9 (1)	12 (1)
**29**	853 (68)	189 (15)	35 (3)	15 (1)	91 (7)	1 (< 1)	31 (3)	10 (1)	14 (1)
**30**	830 (66)	236 (19)	60 (5)	6 (< 1)	56 (4)	< 1 (< 1)	23 (2)	8 (1)	12 (1)
**31**	444 (71)	100 (16)	19 (3)	5 (1)	38 (6)	< 1 (< 1)	9 (1)	3 (< 1)	4 (1)
**32**	2080 (55)	468 (12)	97 (3)	80 (2)	630 (17)	4 (< 1)	199 (5)	45 (1)	95 (3)

No polymer formation was observed. 1-Butene content was calculated using the α-value.

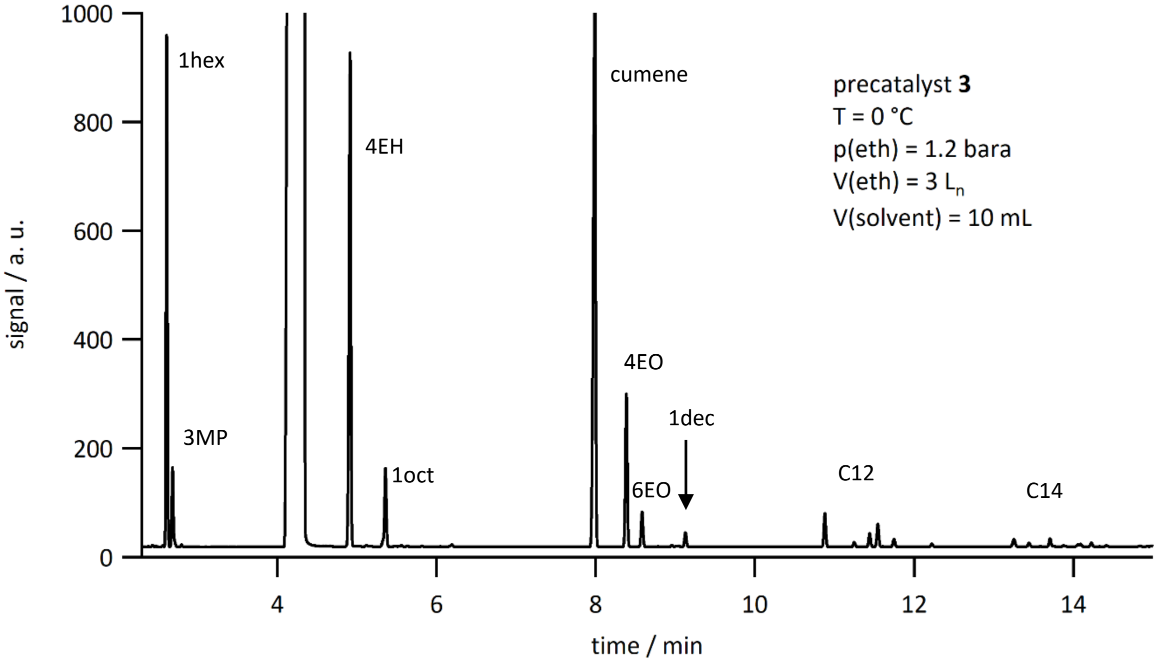

Figure S 13: Gas chromatogram of the products obtained from the ethylene trimerization run at 0 °C and 1.2 bara pressure using precatalyst 3 (table S12, entry 32, 3 L_n ethylene).

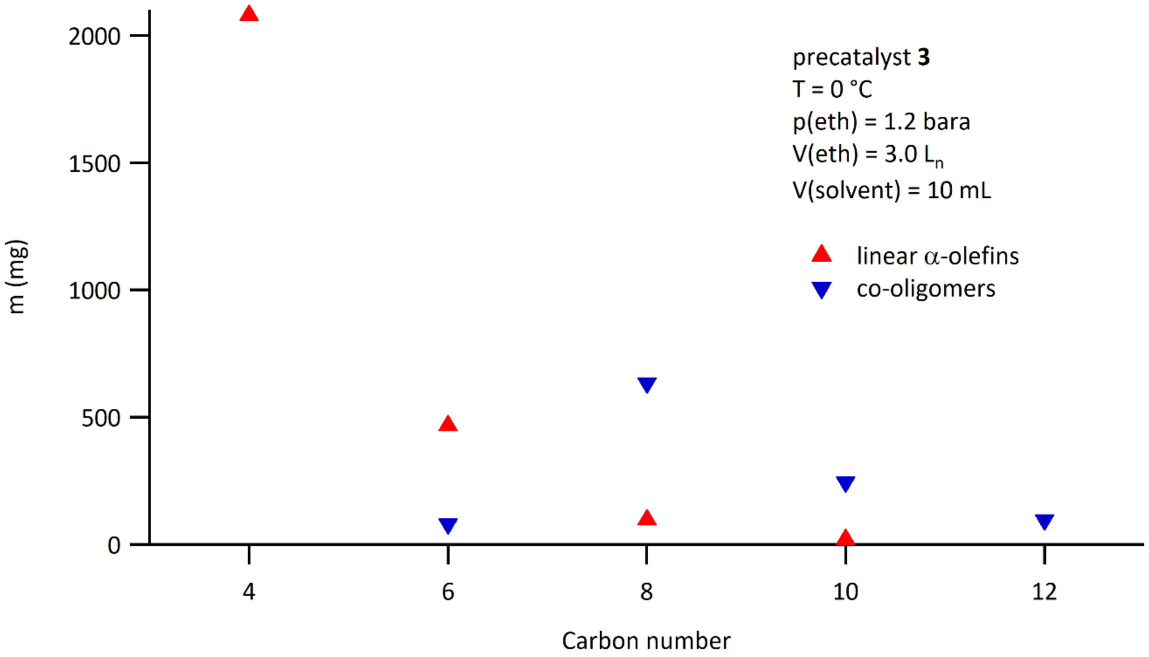

Figure S 14: Plot of detected olefin masses (GC) against the carbon number from ethylene tetramerization (table S 12, entry 32).

Table S 14: Influence of the ethylene consumption on the co-oligomerization of 1-butene and ethylene using precatalyst 3.

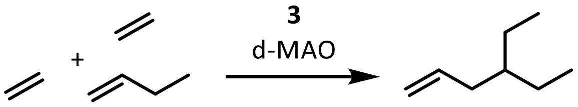

Entry	V(eth) / L_n	Co-oligomers / mol-%				
		3MP	4EH	3MH	4EO+6EO	C12
33	10	7	76	<1	11	4
34	20	7	74	<1	12	5
35	50	7	71	<1	13	6
36	100	7	68	<1	14	7
37	120	8	66	<1	14	8

Reaction conditions: n(Ti) = 0.5 µmol; n(TIBA) = 200 µmol; $\frac{n(dMAO)}{n(Ti)}$ *=250; T = 30 °C; p(eth) = 4.0 bar; V(1-butene) = 200 mL; internal standard: cumene; selectivity based on co-oligomer distribution.*
α(lin. olefins) = 0.21; activity: 9100 $\frac{kg(eth)}{mol\ h\ bar}$.

Table S 15: Overall mass and wt-% of the large-scale co-oligomerization of 1-butene and ethylene using 3 (table S 14).

Entry	m(1But) / g (wt-%)	m(1Hex) / g (wt-%)	m(1Oct) /g (wt-%)	m(3MP) / g (wt-%)	m(4EH) / g (wt-%)	m(3MH) / g (wt-%)	m(4EO) / g (wt-%)	m(6EO) / g (wt-%)	m(C12) / g (wt-%)
33	5.40 (32)	1.70 (10)	0.47 (3)	0.43 (3)	6.54 (39)	0.01 (<1)	0.37 (2)	0.84 (5)	0.57 (3)
34	10.94 (33)	3.45 (10)	1.05 (3)	0.82 (2)	12.26 (37)	0.03 (<1)	0.86 (3)	1.66 (5)	1.23 (4)
35	29.12 (36)	9.17 (11)	2.60 (3)	1.89 (2)	25.96 (32)	0.07 (<1)	2.65 (3)	3.33 (4)	3.29 (4)
36	59.09 (37)	18.61 (12)	5.13 (3)	3.77 (2)	47.82 (30)	0.21 (<1)	6.79 (4)	5.71 (4)	7.43 (5)
37	70.17 (36)	22.10 (11)	5.91 (3)	5.02 (3)	56.81 (29)	0.24 (<1)	9.01 (5)	6.54 (3)	9.93 (5)

No polymer formation was observed. 1-Butene content was calculated using the α-value.

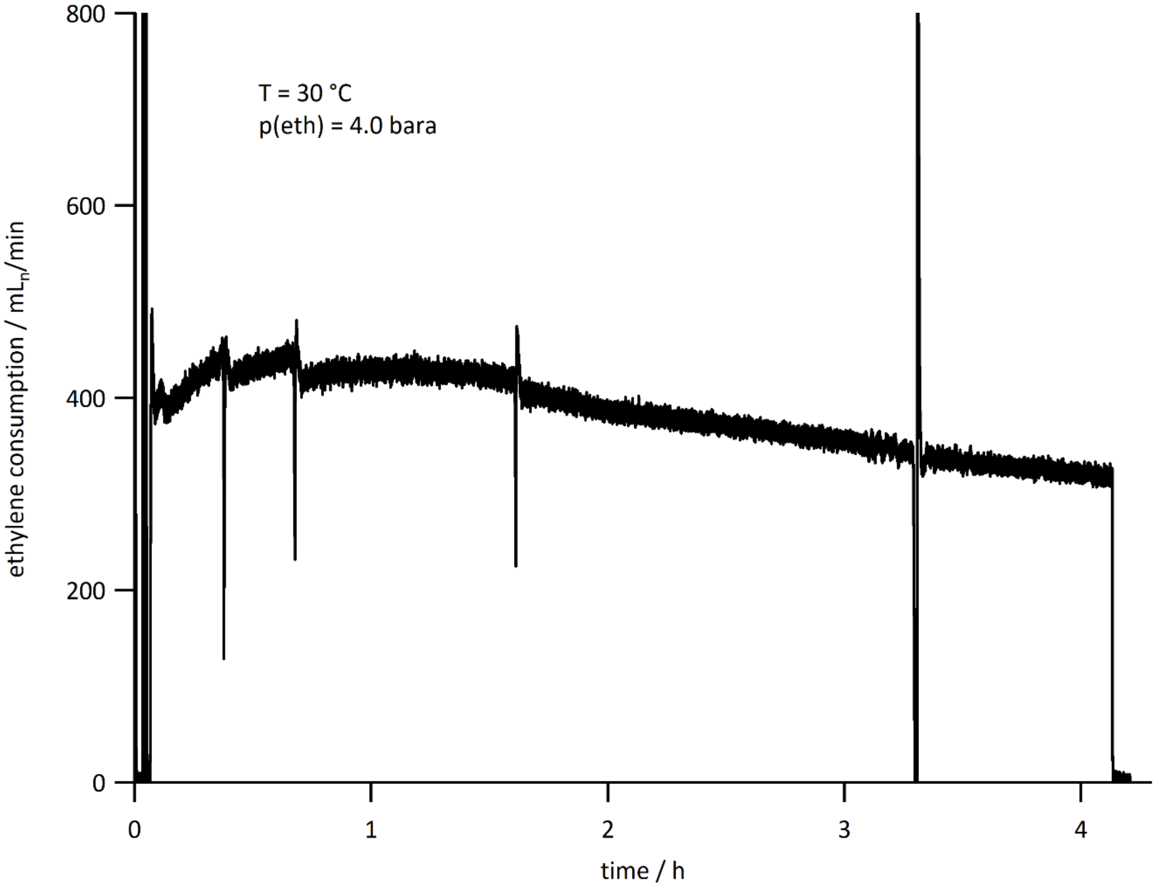

Figure S 15: Ethylene consumption plot of the 1-butene/ethylene co-oligomerization run over several hours using 3 (table S 14 entries 33 – 37). The sharp cuts visible in the ethylene uptake mark points of sample collection. For this purpose, the mechanical stirring was stopped for a short period of time.

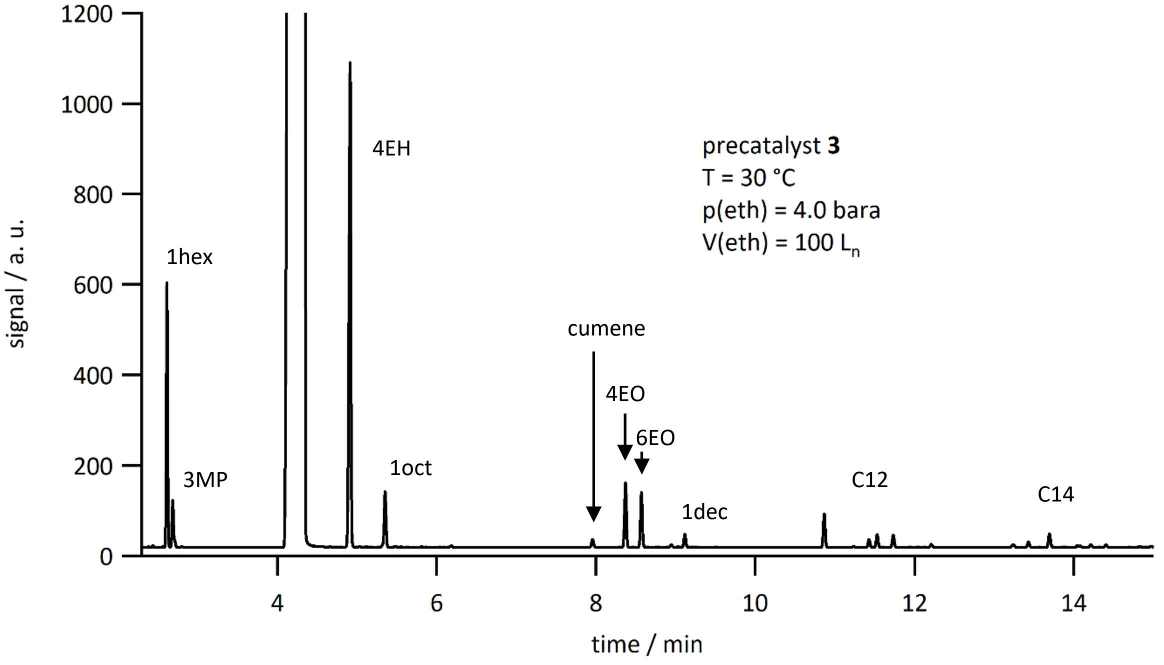

Figure S 16: Gas chromatogram of the products obtained from the 1-butene/ethylene co-oligomerization run at 30 °C and 4.0 bar pressure using precatalyst 3 (table S14, entry 36, 100 L$_n$ ethylene).

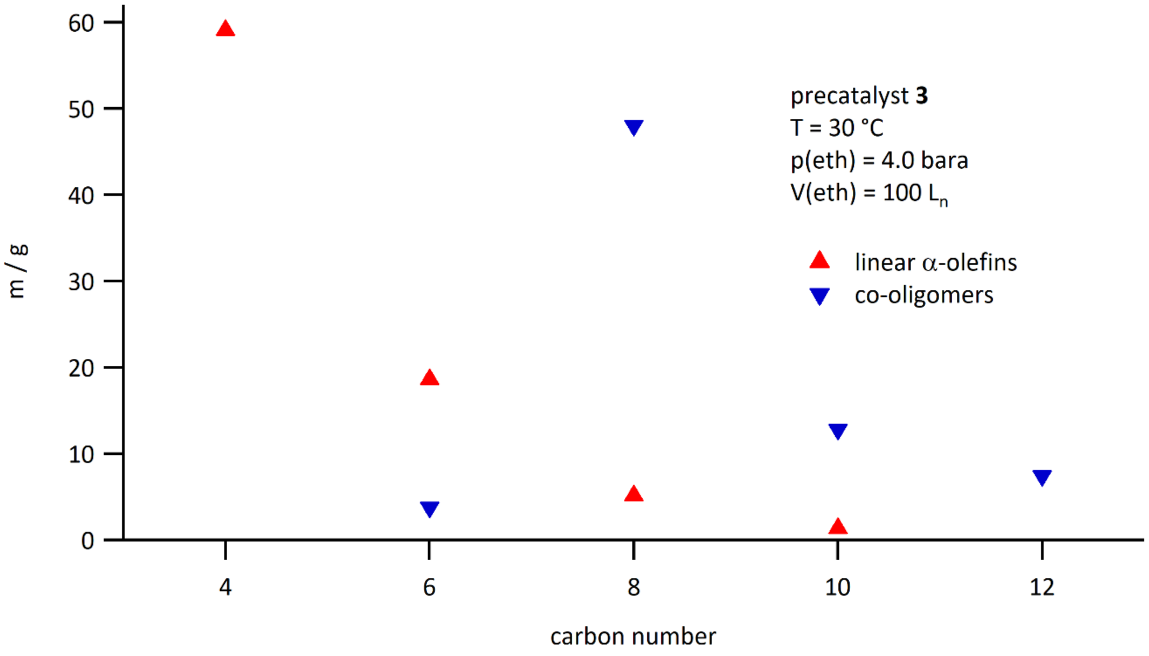

Figure S 17: Plot of detected olefin masses (GC) against the carbon number from the large-scale 1-butene/ethylene co-oligomerization using precatalyst 3 (table S 14, entry 36, 100 L$_n$ ethylene).

Table S 16: Large-scale 1-butene co-oligomerizations at different temperatures using precatalyst 1.

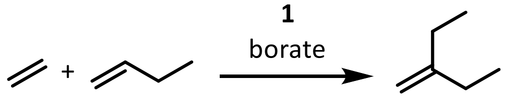

Entry	T / °C	P(eth) / bar	Co-oligomers / mol-%					α	Activity / kg_{Eth} mol_{Ti}^{-1} h^{-1} bar^{-1}
			3MP	4EH	3MH	4EO+6EO	C12		
38	30	4.0	38	40	3	14	4	0.14	5 000
39	0	1.2	53	35	2	6	3	0.08	9 900

Reaction conditions: n(Ti) = 0.5 µmol; n(TIBA) = 200 µmol; $\frac{n(borate)}{n(Ti)}$*=1.1; V(1-butene) = 200 mL; internal standard: cumene; selectivity based on co-oligomer distribution.*

Table S 17: Overall mass and wt-% of the large-scale co-oligomerizations of 1-butene and ethylene using 1 (table S 16).

Entry	m(1But) / g (wt-%)	m(1Hex) / g (wt-%)	m(1Oct) / g (wt-%)	m(3MP) / g (wt-%)	m(4EH) / g (wt-%)	m(3MH) / g (wt-%)	m(4EO) / g (wt-%)	m(6EO) / g (wt-%)	m(C12) / g (wt-%)
38	83.04 (57)	17.44 (12)	4.16 (3)	11.48 (8)	16.06 (11)	1.30 (1)	5.57 (4)	1.51 (1)	6.81 (5)
39	83.62 (52)	9.41 (6)	0.70 (<1)	29.40 (18)	25.78 (16)	1.52 (1)	3.93 (2)	1.39 (1)	3.47 (2)

No polymer formation was observed. 1-Butene content was calculated using the α-value.

References

[1] L. Toldy, M. Kürti and I. Schäfer, *Ger. Pat.,* DE 2916140, **1979** (Egyt Gyo. Gyar.).
[2] T. Dietel, F. Lukas, W. P. Kretschmer, R. Kempe, *Science* **2022**, *375*, 1021-1024.
[3] D. S. McGuinness, A. J. Rucklidge, R. P. Tooze, A. M. Z. Slawin, *Organometallics* **2007**, *26*, 2561-2569.
[4] O. V. Dolomanov, L. J. Bourhis, R. J. Gildea, J. A. K. Howard, H. Puschmann, *J. Appl. Crystallogr.* **2009**, *42*, 339-341.
[5] G. M. Sheldrick, *Acta Crystallogr. Sect. C Struct. Chem.* **2015**, *71*, 3-8.
[6] C. F. MacRae et al. *J. Appl. Cryst.* **2020**, *53*, 226-235.
